# Comparative effectiveness of explainable machine learning approaches for extrauterine growth restriction classification in preterm infants using longitudinal data

**DOI:** 10.3389/fmed.2023.1166743

**Published:** 2023-11-29

**Authors:** Kee Hyun Cho, Eun Sun Kim, Jong Wook Kim, Cheol-Heui Yun, Jae-Won Jang, Payam Hosseinzadeh Kasani, Heui Seung Jo

**Affiliations:** ^1^Department of Pediatrics, Kangwon National University Hospital, Chuncheon, Republic of Korea; ^2^Department of Pediatrics, Kangwon National University School of Medicine, Chuncheon, Republic of Korea; ^3^Department of Computer Science, Sangmyung University, Seoul, Republic of Korea; ^4^Department of Agricultural Biotechnology, Seoul National University, Seoul, Republic of Korea; ^5^Research Institute of Agriculture and Life Sciences, Seoul National University, Seoul, Republic of Korea; ^6^Department of Neurology, Kangwon National University Hospital, Chuncheon, Republic of Korea; ^7^Department of Neurology, Kangwon National University School of Medicine, Chuncheon, Republic of Korea; ^8^Interdisciplinary Graduate Program in Medical Bigdata Convergence, Kangwon National University, Chuncheon, Republic of Korea

**Keywords:** preterm birth, extrauterine growth restriction, machine learning, classification, model trustworthy, interpretability

## Abstract

**Introduction:**

Preterm birth is a leading cause of infant mortality and morbidity. Despite the improvement in the overall mortality in premature infants, the intact survival of these infants remains a significant challenge. Screening the physical growth of infants is fundamental to potentially reducing the escalation of this disorder. Recently, machine learning models have been used to predict the growth restrictions of infants; however, they frequently rely on conventional risk factors and cross-sectional data and do not leverage the longitudinal database associated with medical data from laboratory tests.

**Methods:**

This study aimed to present an automated interpretable ML-based approach for the prediction and classification of short-term growth outcomes in preterm infants. We prepared four datasets based on weight and length including weight baseline, length baseline, weight follow-up, and length follow-up. The CHA Bundang Medical Center Neonatal Intensive Care Unit dataset was classified using two well-known supervised machine learning algorithms, namely support vector machine (SVM) and logistic regression (LR). A five-fold cross-validation, and several performance measures, including accuracy, precision, recall and F1-score were used to compare classifier performances. We further illustrated the models’ trustworthiness using calibration and cumulative curves. The visualized global interpretations using Shapley additive explanation (SHAP) is provided for analyzing variables’ contribution to final prediction.

**Results:**

Based on the experimental results with area under the curve, the discrimination ability of the SVM algorithm was found to better than that of the LR model on three of the four datasets with 81%, 76% and 72% in weight follow-up, length baseline and length follow-up dataset respectively. The LR classifier achieved a better ROC score only on the weight baseline dataset with 83%. The global interpretability results revealed that pregnancy-induced hypertension, gestational age, twin birth, birth weight, antenatal corticosteroid use, premature rupture of membranes, sex, and birth length were consistently ranked as important variables in both the baseline and follow-up datasets.

**Discussion:**

The application of machine learning models to the early detection and automated classification of short-term growth outcomes in preterm infants achieved high accuracy and may provide an efficient framework for clinical decision systems enabling more effective monitoring and facilitating timely intervention.

## 1 Introduction

Preterm infants are increasingly being diagnosed with extrauterine growth restriction (EUGR). EUGR refers to insufficient growth during hospitalization and represents a significant clinical challenge globally, particularly in preterm infants. The inadequate growth of EUGR can extend beyond the hospitalization period and have both short- and long-term implications, including growth failure (1), adverse neurodevelopmental outcomes (2), and cardiovascular risk (3). According to their clinical circumstances, preterm newborns currently constitute a large and heterogeneous population. Premature birth is a leading cause of long-term neurodevelopmental difficulties and disabilities (4). According to the World Health Organization, 15 million infants are delivered prematurely each year throughout the world (5). However, the incidence of EUGR has been reported to jeopardize 40 to 95% of premature infants (6). Typically, EUGR is diagnosed when the newborn’s weight is <10th percentile at either discharge or 36 to 40 weeks postmenstrual age (7, 8). In fact, statistics published by Clark et al. revealed significant EUGR results in terms of weight (28%), length (34%), and head circumference (16%) (9) in preterm infants during hospitalization. Furthermore, the National Institute of Child and Human Development (NICHD), has reported that the prevalence of postnatal growth failure in preterm infants with very low birth weight admitted to neonatal intensive care units (NICU) is approximately 89% (10), which may further worsen the prognosis. EUGR is multifactorial in etiology, in which both genetic and environmental factors play a role, and it potentially exposes preterm infants to multiple morbidities. Notably, several studies have linked poor postnatal growth to an increased morbidity and mortality both in the neonatal period and in later life (11–14).

Assessing and monitoring the physical growth of infants is fundamental to effective treatment of EUGR, which can potentially reduce the escalation of this disorder. However, early evaluation and recognition of EUGR should be emphasized when caring for preterm infants, given the similarities in the clinical manifestations, especially in extremely premature newborns. Therefore, the development of optimal strategies for early diagnosis of clinical deterioration based on longitudinal data is necessary. Ongoing research is focused on developing data mining strategies to improve the understanding of the underlying disease processes. Biomarkers, including clinical symptoms, laboratory results and imaging modalities, play a critical role in this regard. As laboratory testing is the backbone of clinical decision-making, its application in medicine is quite promising. The application of ML algorithms may deliver insights that help healthcare systems diagnose and treat these diseases early. Consequently, the use of ML in laboratory medicine is gaining popularity and becoming increasingly vital for clinicians (15, 16). Despite significant improvements in neonatal care over the past two decades that have led to better survival rates and reduced complications in preterm infants, growth restriction remains a common issue during the postnatal period (17). As a result, there is an urgent need for novel approaches to reduce the risks associated with EUGR (18). Consequently, the attention of healthcare professionals is shifting to preventive strategies based on prospective longitudinal studies with long-term follow-up care and is not limited to cross-sectional measurements. However, obtaining long-term follow-up data can be challenging, and such data remain scarce (19, 20). Over the previous few decades, considerable advances have been made in health data generation and collection, particularly in terms of clinical information (21). This health record information may contain personal disease histories, diagnosis mechanisms, treatment processes, and hospital administration information to provide statistical background for epidemiological records (22, 23). It is of great value to discover hidden patterns in this information.

Given the current pace of artificial intelligence (AI) development in medical fields, many healthcare systems need an evidence-based approach based on longitudinal-oriented data to realize automated analysis, which may facilitate treatment planning and decision-making processes (24, 25). Therefore, accurate computer-aided diagnosis (CAD) methods can help clinicians to discover hidden patterns in data. CAD system are being extensively used in healthcare (26). Despite the significant advances in AI in medical fields, the field of pediatrics has been slow to adopt these technologies. AI-based predictive analysis incorporates a variety of ML algorithms and data mining techniques that use data to predict future events. ML is a powerful automated analysis technique and subfield of AI, which uses computer algorithms and has been successfully employed in clinical applications for classification, prediction, and decision-making in a multitude of disciplines (27–30). To address the above-mentioned issues, this study aimed to develop a ML system capable of accurately predicting EUGR and identifying the clinical risk factors associated with EUGR in preterm infants.

The use of machine learning algorithms in neonatal care has been gaining attraction in recent years, with several studies demonstrating the effectiveness of these techniques in predicting various outcomes. For instance, Han et al. (31) aimed to predict postnatal growth failure (PGF) among very low birth weight (VLBW) infants using machine learning models. They compared four different techniques [extreme gradient boosting (XGB), random forest, support vector machine, and convolutional neural network] against the conventional multiple logistic regression (MLR) model. The XGB algorithm showed the best performance, with a 74% area under the receiver operating characteristic curve (AUROC) and 68% accuracy for Day 7 compared to MLR. The authors concluded that machine learning algorithms, particularly XGB, could help neonatologists identify high-risk infants for PGF and enable early intervention. Leigh et al. (32) applied machine learning to predict bronchopulmonary dysplasia (BPD)-free survival among very preterm infants using data from 689 infants. The final model demonstrated 92.10% receiver operating characteristics performance in both the training and validation datasets. The study suggested that machine learning-based BPD prediction, considering perinatal features and respiratory data, may have clinical applicability for early targeted intervention in high-risk infants. Wu et al. (33) conducted a retrospective cohort study to predict late respiratory support in preterm infants using machine learning algorithms. They collected data on very-low-birth-weight infants born between 2016 and 2019 from the Taiwan Neonatal Network database. Logistic regression yielded the 88.10% (AUROC) overall mortality. The authors concluded that machine learning could be used to develop models for predicting late respiratory support, with simplified estimators for clinical application. Additionally, Podda et al. (34) developed the Preterm Infants Survival Assessment (PISA) predictor using machine learning methods, specifically artificial Neural Networks (NN), on a cohort of neonates with gestational age <30 weeks or birth weight <1,501 g. The resulting predictor was compared with logistic regression models, and the NN approach showed (91.49%) a small but significant advantage over logistic regression 91.47% approaches. These studies collectively highlight the potential for machine learning to improve outcomes in neonatal care and enable early targeted intervention for high-risk infants.

However, only a few published articles on laboratory test biomarkers exists, and there is a lack of measuring these biomarkers in a follow-up manner, in which infants’ data are tracked after a period of time. These limitations underscore the need for more research in this area to improve our understanding of the underlying disease processes and enable early diagnosis of clinical deterioration. Given the potential benefits of ML algorithms in predicting outcomes and enabling early intervention in neonatal care, we aim to conduct a comprehensive analysis of the applications of ML techniques using a longitudinal approach. In addition, we aim to conduct a global interpretation to identify the most important variables during each time period, providing a more comprehensive understanding of the factors that contribute to growth failure in premature infants. Specifically, by generating four datasets, two each for weight and length outcomes, for both baseline and follow-up measurements, we aim to investigate potential risk factors for growth failure and examine how these factors change over time. The use of standardized growth charts and the longitudinal approach will enable more accurate comparisons and provide a comprehensive understanding of the factors that contribute to growth failure in premature infants. This comprehensive analysis of potential risk factors for growth failure among preterm infants utilizes a longitudinal approach that tracks changes in these factors over time, allowing for better insights into the underlying mechanisms affecting growth.

The remainder of the paper is structured as follows. Section 2 presents the data used in the research and describes the preprocessing and classification algorithms. Next, Section 3 presents the experimental results of this study, and the discussion is presented in Section 4. Finally, the conclusions are found in Section 5.

## 2 Materials and methods

### 2.1 Data description

A single-center prospective observational cohort study was conducted in which infants received either fortified breast milk or preterm formula in a level 3 NICU in South Korea. All infants admitted to the NICU of CHA Bundang Medical Center were eligible for participation if their gestational age was less than 34 weeks or their birth weight was less than 1,500 g. The gestational age was determined based on the menstrual history and antenatal ultrasound, or by physical examination if discrepancies were present. The exclusion criteria were the presence of a major congenital anomaly, gastrointestinal tract disorder, or failure to commence enteral feeding within 7 days of life. Infants were assigned to the fortified-breast-milk-fed (BM) or premature-formula-fed (PM) groups according to their initial analysis results. After exclusion, we included 124 premature infants in this study. The collected data with the same race and ethnicity included demographic data and the initial assessment results (including vital signs, imaging findings, and laboratory tests). We employed 26 predictor variables (independent variables) and target outcome as input to ML models. These predictors were carefully selected based on their relevance to the EUGR as target outcome. The 22 predictor variables cover a range of factors that potentially influence the occurrence of EUGR. The infants who have genetic diseases or whose mothers have genetic diseases during the recruitment phase, similar to patients with significant congenital anomalies were excluded. All the medications administered to the enrolled infants included surfactant, antibiotics, intravenous immunoglobulin, granulocyte colony-stimulating factor(G-CSF), caffeine citrate, ibuprofen, calcium gluconate, and prophylactic antifungal agents, as clinically indicated. Additionally, during the period of inadequate enteral nutrition, total parenteral nutrition was delivered, which was subsequently followed by the provision of multivitamins and additional vitamin D supplementation. Notably, no detectable adverse effects were observed throughout the study period. The demographic attributes included the gestational age, sex, twin, weight at birth, length at birth, and head circumference at birth. The maternal characteristic attributes included the maternal height and maternal body mass index. The perinatal characteristic attributes included assisted reproductive technologies such as *in vitro* insemination (IVF) and intrauterine insemination (IUI), gestational diabetes mellitus (GDM), pregnancy-induced hypertension (PIH), antenatal corticosteroids (ANC), premature rupture of the membrane (PROM) ≥ 18 h, cesarean section, and APGAR (Appearance, Pulse, Grimace, Activity, and Respiration) score at 1–5 min. The neonatal characteristic attributes included respiratory distress syndrome of newborns (RDS), hemodynamically significant patent ductus arteriosus (hsPDA), duration of positive pressure ventilation (PPV), duration of oxygen supply, types of feeding, and days to full feeding. This study was approved by the Institutional Review Board of the CHA Bundang Medical Center (BD2015-223). The data were prospectively collected after informed consent was obtained from all participants.

This study focuses on two primary outcomes, extrauterine growth restriction by weight and length, using the 25th percentile as a cutoff for proper growth. Growth percentiles for weight and length were calculated using the Fenton preterm growth chart (35), which takes into account the gestational age and sex of the infant at birth for infants born between 22 and 40 weeks of gestation. The Fenton growth chart is a widely accepted tool for assessing preterm infant growth and provides a standardized means of evaluating growth percentiles, enabling more accurate comparisons across different populations and research studies. Four datasets were generated for baseline and follow-up measurements, including two datasets for baseline (weight-baseline and length-baseline) and two follow-up datasets (weight follow-up and length follow-up). While all datasets use the same predictor variables, their values differ as time passes during the follow-up period. For the baseline datasets, the outcome variable in the weight-based dataset is the infant’s weight at baseline, while the outcome variable in the length-based dataset is the infant’s length at baseline. For the follow-up datasets, the outcome variable in the weight-based dataset is the infant’s weight at follow-up, while the outcome variable in the length-based dataset is the infant’s length at follow-up.

The dataset descriptions for weight and length are provided in Tables 1, 2, respectively. These tables provide a detailed overview of the datasets used in the study, including the number of subjects, the distribution of EUGR cases, and the key features used in the ML models. The dataset descriptions are an important reference for in replicating results or applying similar ML approaches to datasets.

**Table 1:** Demographic and laboratory test characteristics of the subjects in the baseline dataset.

	Baseline length dataset		Baseline weight dataset		
Diagnosis	EUGR (*n* = 35)	Non-EUGR (*n* = 89)	EUGR (*n* = 29)	Non-EUGR (*n* = 95)	Missing values (%)
GA	32.02 ± 2.06	31.31 ± 2.45	32.55 ± 1.76	31.20 ± 2.43	-
Birth weight	1471.29 ± 391.32	1697.70 ± 457.33	1434.31 ± 294.84	1694.68 ± 472.20	-
Birth length	38.40 ± 2.90	41.36 ± 3.72	39.71 ± 3.03	40.77 ± 3.92	-
**Sex**					-
Male	20 (57.14%)	52 (58.43%)	17 (58.62%)	55 (57.89%)	-
Female	15 (42.86%)	37 (41.57%)	12 (41.38%)	40 (42.11%)	-
**Twin**					-
Singleton	21 (60.00%)	49 (55.06%)	15 (51.72%)	55 (57.89%)	-
Twin	14 (40.00%)	40 (44.94%)	14 (48.28%)	40 (42.11%)	-
Maternal age	33.23 ± 3.57	32.37 ± 4.43	33.41 ± 4.75	32.37 ± 4.02	-
Maternal BMI	22.20 ± 2.88	23.85 ± 3.71	23.44 ± 2.86	23.37 ± 3.76	-
**GDM**					-
No	30 (85.71%)	74 (83.15%)	24 (82.76%)	80 (84.21%)	-
Yes	5 (14.29%)	15 (16.85%)	5 (17.24%)	15 (15.79%)	-
**PIH**					-
No	21 (60.00%)	80 (89.89%)	15 (51.72%)	86 (90.53%)	-
Yes	14 (40.00%)	9 (10.11%)	14 (48.28%)	9 (9.47%)	-
**ANC**					-
Yes	22 (62.86%)	60 (67.42%)	24 (82.76%)	58 (61.05%)	-
No	13 (37.14%)	29 (32.58%)	5 (17.24%)	37 (38.95%)	-
**PROM**					-
No	32 (91.43%)	73 (82.02%)	29 (100.00%)	76 (80.00%)	-
Yes	3 (8.57%)	16 (17.98%)	0 (0.00%)	19 (20.00%)	-
**Csec**					-
Yes	30 (85.71%)	76 (85.39%)	25 (86.21%)	81 (85.26%)	-
No	5 (14.29%)	13 (14.61%)	4 (13.79%)	14 (14.74%)	-
5APGAR	7.77 ± 1.03	7.79 ± 1.06	7.90 ± 1.01	7.75 ± 1.06	-
WBC	5971.10 ± 2307.99	8642.44 ± 5774.19	6184.08 ± 2441.38	8408.71 ± 5665.85	7.26
Hb	16.42 ± 1.63	14.96 ± 1.83	16.46 ± 1.92	15.04 ± 1.75	7.26
CRP	0.03 ± 0.06	0.07 ± 0.24	0.02 ± 0.04	0.07 ± 0.23	7.26
Albumin	3.25 ± 0.27	3.08 ± 0.29	3.23 ± 0.30	3.10 ± 0.28	7.26
BUN	11.86 ± 7.24	19.06 ± 98.42	11.66 ± 7.15	18.66 ± 95.26	7.26
Cr	0.61 ± 0.20	0.50 ± 0.17	0.64 ± 0.21	0.50 ± 0.16	7.26
Ca	9.21 ± 0.96	9.42 ± 0.72	9.13 ± 0.86	9.43 ± 0.77	7.26
P	5.92 ± 0.81	5.93 ± 0.93	6.01 ± 0.89	5.90 ± 0.90	7.26
Mg	3.16 ± 1.09	3.06 ± 0.92	3.49 ± 1.15	2.97 ± 0.87	22.58
VitD	18.54 ± 8.75	20.14 ± 7.40	18.00 ± 8.45	20.20 ± 7.56	12.90
BM	0.00 ± 0.00	0.00 ± 0.00	0.00 ± 0.00	0.00 ± 0.00	-
**Invasive MV**					-
Yes	22 (62.86%)	57 (64.04%)	16 (55.17%)	63 (66.32%)	-
No	13 (37.14%)	32 (35.96%)	13 (44.83%)	32 (33.68%)	-
**PPV**					-
Yes	29 (82.86%)	67 (75.28%)	22 (75.86%)	74 (77.89%)	-
No	6 (17.14%)	22 (24.72%)	7 (24.14%)	21 (22.11%)	-

^1^Data are presented as the mean ± SD values except were indicated; *n*/*N* (%), the figures in parentheses are percentages. n, number of participants; EUGR, extrauterine growth restriction; BL, birth length; BWt, birth weight; GA, gestational age; BMI, body mass index; GDM, gestational diabetes mellitus; PIH, pregnancy-induced hypertension; ANC, antenatal corticosteroid; PROM, premature rupture of membrane; Csec, Cesarean section; 5APGAR, Apgar score at 5 min.; WBC, white blood cells; Hb, hemoglobin; CRP, C-reactive protein; BUN, blood urea nitrogen; Cr, creatinine; Ca, calcium; P, phosphorous; Mg, magnesium; Vit D, vitamin D; MV, mechanical ventilation; PPV, positive pressure ventilation.

**Table 2:** Demographic and laboratory test characteristics of the subjects in the follow-up dataset.

	Follow-up length dataset		Follow-up weight dataset		
Diagnosis	EUGR (*n* = 83)	Non-EUGR (*n* = 41)	EUGR (*n* = 72)	Non-EUGR (*n* = 52)	Missing values (%)
GA	31.27 ± 2.47	31.85 ± 2.17	31.31 ± 2.48	31.93 ± 2.05	-
Birth weight	1485.76 ± 421.04	1838.75 ± 409.47	1483.43 ± 392.15	1938.17 ± 406.65	-
Birth length	39.28 ± 3.46	42.24 ± 3.46	39.52 ± 3.54	42.56 ± 3.34	-
**Sex**					-
Male	40 (55.56%)	32 (61.54%)	49 (59.04%)	23 (56.10%)	-
Female	32 (44.44%)	20 (38.46%)	34 (40.96%)	18 (43.90%)	-
**Twin**					-
Singleton	38 (52.78%)	32 (61.54%)	42 (50.60%)	28 (68.29%)	-
Twin	34 (47.22%)	20 (38.46%)	41 (49.40%)	13 (31.71%)	-
Maternal age	32.83 ± 3.94	32.31 ± 4.57	32.89 ± 4.05	32.05 ± 4.52	-
Maternal BMI	23.34 ± 3.71	23.44 ± 3.38	22.81 ± 3.04	24.55 ± 4.25	-
**GDM**					-
No	58 (80.56%)	46 (88.46%)	70 (84.34%)	34 (82.93%)	-
Yes	14 (19.44%)	6 (11.54%)	13 (15.66%)	7 (17.07%)	-
**PIH**					-
No	53 (73.61%)	48 (92.31%)	63 (75.90%)	38 (92.68%)	-
Yes	19 (26.39%)	4 (7.69%)	20 (24.10%)	3 (7.32%)	-
**ANC**					-
Yes	45 (62.50%)	37 (71.15%)	53 (63.86%)	29 (70.73%)	-
No	27 (37.50%)	15 (28.85%)	30 (36.14%)	12 (29.27%)	-
**PROM**					-
No	63 (87.50%)	42 (80.77%)	73 (87.95%)	32 (78.05%)	-
Yes	9 (12.50%)	10 (19.23%)	10 (12.05%)	9 (21.95%)	-
**Csec**					-
Yes	65 (90.28%)	41 (78.85%)	76 (91.57%)	30 (73.17%)	-
No	7 (9.72%)	11 (21.15%)	7 (8.43%)	11 (26.83%)	-
5APGAR	7.82 ± 1.04	7.73 ± 1.07	7.73 ± 1.04	7.88 ± 1.08	-
WBC	9672.26 ± 1970.56	8979.25 ± 2022.27	9775.25 ± 1944.22	8584.82 ± 1935.97	16.94
Hb	10.38 ± 1.00	10.64 ± 1.24	10.37 ± 1.06	10.73 ± 1.18	17.74
CRP	0.06 ± 0.07	0.07 ± 0.14	0.07 ± 0.12	0.05 ± 0.04	18.55
Albumin	3.44 ± 0.18	3.38 ± 0.18	3.42 ± 0.18	3.40 ± 0.18	16.33
BUN	5.26 ± 1.46	5.59 ± 1.46	5.33 ± 1.55	5.55 ± 1.27	16.33
Cr	0.31 ± 0.08	0.33 ± 0.09	0.31 ± 0.08	0.33 ± 0.09	16.33
Ca	10.35 ± 0.35	10.25 ± 0.22	10.32 ± 0.33	10.29 ± 0.24	14.52
P	6.46 ± 0.50	6.44 ± 0.42	6.42 ± 0.50	6.51 ± 0.38	14.52
Mg	2.03 ± 0.09	2.05 ± 0.10	2.04 ± 0.11	2.05 ± 0.05	34.63
VitD	25.71 ± 5.61	22.86 ± 6.86	24.53 ± 6.36	24.47 ± 6.25	14.52
BM	0.60 ± 0.34	0.62 ± 0.34	0.66 ± 0.31	0.51 ± 0.38	-
**Invasive MV**					-
No	70 (97.22%)	49 (94.23%)	80 (96.39%)	39 (95.12%)	-
Yes	2 (2.78%)	3 (5.77%)	3 (3.61%)	2 (4.88%)	-
**PPV**					-
No	59 (81.94%)	47 (90.38%)	68 (81.93%)	38 (92.68%)	-
Yes	13 (18.06%)	5 (9.62%)	15 (18.07%)	3 (7.32%)	-

^1^Data are presented as the mean ± SD values except were indicated; *n*/*N* (%), the figures in parentheses are percentages. n, number of participants; EUGR, extrauterine growth restriction; BL, birth length; BWt, birth weight; GA, gestational age; BMI, body mass index; GDM, gestational diabetes mellitus; PIH, pregnancy-induced hypertension; ANC, antenatal corticosteroid; PROM, premature rupture of membrane; Csec, Cesarean section; 5APGAR, Apgar score at 5 min.; WBC, white blood cells; Hb, hemoglobin; CRP, C-reactive protein; BUN, blood urea nitrogen; Cr, creatinine; Ca, calcium; P, phosphorous; Mg, magnesium; Vit D, vitamin D; MV, mechanical ventilation; PPV, positive pressure ventilation.

### 2.2 Data preprocessing

The test results of the classifier depend heavily on the background knowledge of the sample data. Therefore, it is of great importance to preprocess the sample raw data to acquire an effective classification performance. Data normalization is a crucial preprocessing step that involves scaling or transforming the data before evaluating it with machine learning algorithms (31). In this study, we addressed the missing values in the dataset by employing the mean imputation technique where the missing values are replaced with the mean value of the corresponding feature. Mean imputation is a widely-used approach for handling missing data that preserves the mean and variance of the original data. Additionally, we also applied the MinMaxScaler technique for data normalization. This method scales each feature to a range between 0 and 1, by subtracting the minimum value and dividing by the range of the feature.

### 2.3 Machine learning

**Figure 1:**
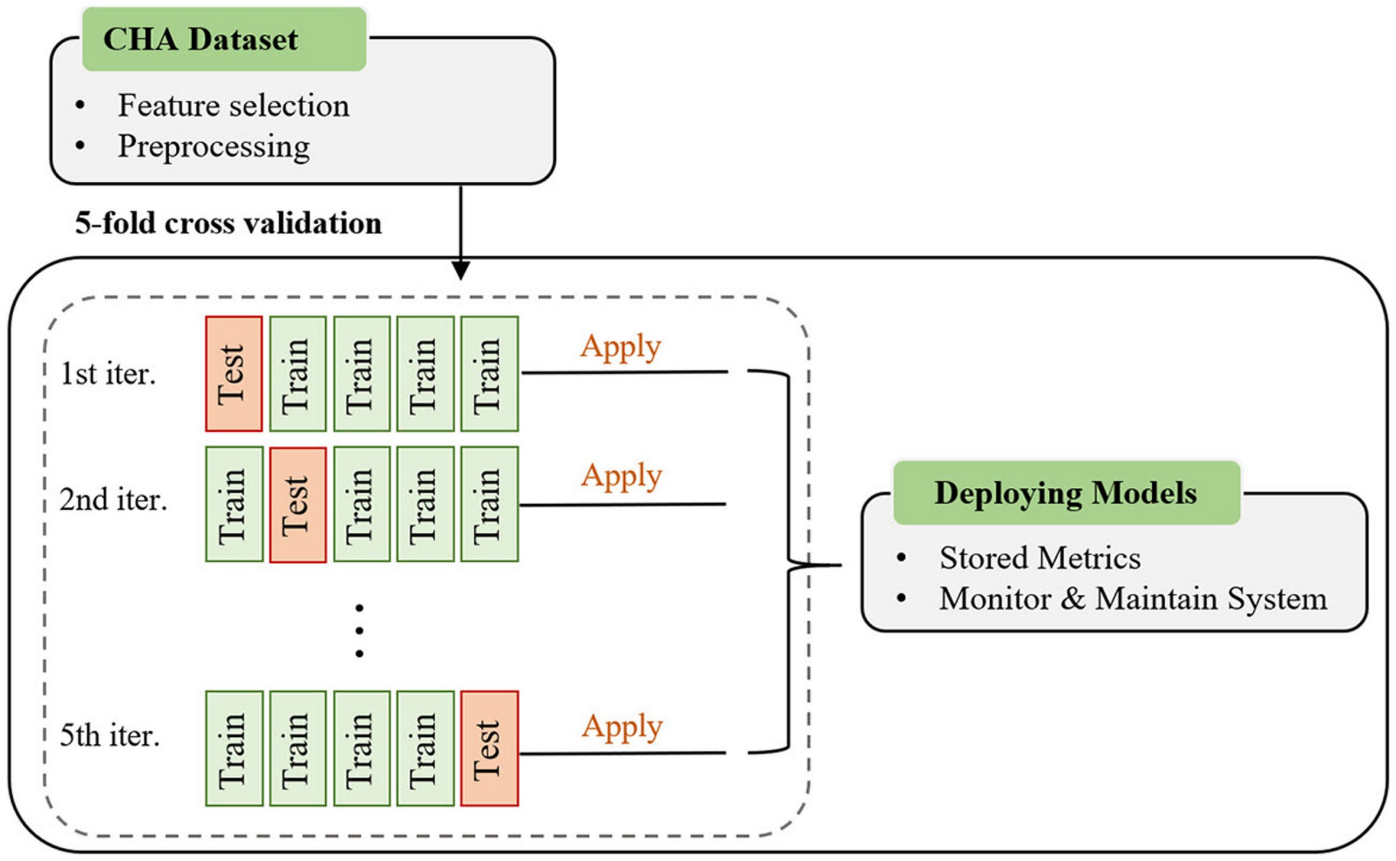
Flowchart of the five-fold cross-validation of the proposed machine learning method.

The performance of different classification algorithms used for classifying non-EUGR and EUGR infants was tested. In many studies, authors often used two validation methods, namely the hold-out and k-fold cross-validation methods to evaluate the capabilities of a model. Cross-validation is a standard method for testing models when datasets are too small to be split into training and test sets (36). Based on the size of dataset, a five k-fold cross-validation method without repetition was used to evaluate the proposed model. The input data were randomly split into five subsets of approximately equal size. During each run, for each subset, the classifier was trained on k-1 folds and then its performance was validated on data in the k-th fold. The final result is the average of all test performances of all folds. For each fold, the area under the curve (AUC) was estimated in both the training and test set. This step is critical to avoid overfitting the classifier to a single training set and ensure that the training and testing datasets are evenly distributed. We used two well-known classifiers, logistic regression (LR) and support vector machine (SVM) to perform classification and build the risk assessment model. To enable a direct and unbiased comparison between the SVM and LR models, the default settings were employed, refraining from the implementation of hyperparameter tuning. In the SVM model, these default settings encompassed the utilization of a radial basis function (RBF) kernel, a regularization parameter (C) set to 1.0, and the automatic estimation of the kernel’s scaling parameter (gamma) based on the characteristics of the dataset. Similarly, the default settings for the LR model involved L2 regularization with an inverse regularization strength (C) of 1.0 and the application of the ‘lbfgs’ solver. The decision to avoid hyperparameter tuning in this study was supported by several factors. Firstly, it ensured a fair and unbiased comparison between the SVM and LR models, eliminating potential biases introduced by inconsistent tuning processes. Secondly, it saved computational resources and time, allowing focus on other critical aspects of the research. Lastly, default settings are often carefully chosen by experts, providing reasonable configurations for a wide range of applications. Thus, refraining from hyperparameter tuning facilitated a straightforward comparison while leveraging the expertise embedded in the default settings of the models. The workflow of proposed methodology is depicted in Figure 1.

#### 2.3.1 Logistic regression

Logistic regression, another technique from the field of statistics borrowed by machine learning, involves modeling the probability of a discrete outcome given an input variable (37). The outcome is measured using a dichotomous variable. LR involves the transformation of linear regression using the sigmoid function, where it gets a linear combination of variables and then applies them to a non-linear sigmoidal function. It is a valuable analysis method for classification problems compared to a regression model as it tries to obtain reliable performance with linearly separable classes and can also be generalized to multiclass classification.

#### 2.3.2 Support vector machine

This method involves determining the class of data points using appropriate hyperplanes in a multidimensional space (38). By using SVM, we aim to find a hyperplane that separates cases of two categories of variables that take up neighboring clusters of vectors on the other. Support vectors are those that are closer to the hyperplane. Training data is categorized into target values and attributes, and it produces a model for predicting target values for test data.

### 2.4 Evaluation criteria

Model evaluation is an essential component of a classification task. To perform a fair comparison between the classifiers and measure the prediction performance of the ML models, several evaluation metrics including the accuracy (*ACC*), recall (*REC*), precision (*PREC*), F1-score (*F1*), the area under the receiver operating characteristic curve (*AUROC*), and the area under the precision–recall curve (*AUPRC*) were used. The predictive values are also demonstrated in a two-by-two confusion matrix. In this text, true positive (TP) refers to an EUGR positive outcome where the model correctly predicts the positive class, false negative (FN) refers to a non-EUGR infant in which the model incorrectly predicts the EUGR positive class, true negative (TN) refers to a non-EUGR infant where the model correctly predicts the non-EUGR class, and false positive (FP) refers to a non-EUGR infant where the model incorrectly predicts the EUGR positive class. Given TP, TN, FP and FN data, all evaluation metrics were calculated as follows.

Accuracy is the ratio between the overall correctly predicted samples and the total number of examples in the evaluation dataset.


$$ACC=\frac{\mathrm{Correctly}\;\mathrm{classifieds}\;\mathrm{amples}}{\mathrm{All}\;\mathrm{samples}}=\frac{TP+TN}{TP+FP+TN+FN}$$

Recall, also known as the sensitivity or true positive rate (TPR), is the ratio between correctly predicted positive cases from all the samples assigned to the actual positive cases.


$$REC=\frac{\mathrm{True}\;\mathrm{positive}\;\mathrm{samples}}{\mathrm{Samples}\;\mathrm{classified}\;\mathrm{positive}}=\frac{TP}{TP+FN}$$

Precision is the ratio between correctly positive predicted samples concerning all samples assigned to the positive class.


$$\mathrm{PREC}=\frac{\mathrm{Samples}\;\mathrm{correctly}\;\mathrm{classified}}{\mathrm{Samples}\;\mathrm{assigned}\;to\;\mathrm{class}}=\frac{TP}{TP+FP}$$

F1-score is generally defined as the harmonic mean of precision and recall, which penalizes extreme values of either.


$$F1=2\times \frac{\mathrm{Precision}\times \mathrm{recall}\;}{\mathrm{Precision}\times \mathrm{recall}}\;=\frac{2\times TP}{2\times TP+FP+FN}$$

The receiver operating characteristic (ROC) curve is a valuable metric that shows the performance of a classification model at all classification thresholds (39). It is widely used in binary classification and has two parameters. The area under the precision–recall curve (AUPRC) is a valuable metric for classifying imbalanced data (40). We further illustrated the models’ trustworthiness using calibration and cumulative curves and used global interpretations using Shapley additive explanation (SHAP) (41) for analyzing variables’ contribution to final prediction we employed the Shapley Additive Explanations (SHAP) methodology to establish a ranking of the feature importance in our models. SHAP provides each feature with an importance score for a given prediction, and this is done by relying on principles derived from cooperative game theory. Upon training the models, the SHAP Python library applied to calculate the SHAP values for every feature in datasets. This was done by using the KernelExplainer and LinearExplainer from the SHAP library, which is suitable for SVM and LR models, respectively. A SHAP value illustrates the influence a feature has on shifting the prediction, with these values summing up to the difference between the predicted outcome and the base (expected) outcome. By taking the mean of the absolute SHAP values for each feature across all instances, we were able to obtain a measure of global feature importance. Higher SHAP values signify a more important feature, as these contribute more to the prediction outcome. We then ranked the features according to their average absolute SHAP values, thereby gaining a comprehensive understanding of feature importance.

### 2.5 Statistical analysis

The programming work for this study was performed in the Python programming language (version 3.9) (42). All data preprocessing and analysis were carried out using Pandas (43) and NumPy (44), Python libraries for data manipulation and analysis, and Scikit-learn (45), a Python module integrating a wide range of machine learning algorithms. We performed all analyses on 24-core Intel(R) Xeon(R) Gold 5,118 CPU @ 2.30GHz, RAM 128 GB (Intel Corporation, Santa Clara, CA, United States) running Windows 10 Pro.

## 3 Experiment and results

### 3.1 Study population characteristics

This prospective observational study included 124 cases of preterm infants classified as non-EUGR and EUGR based on their weight percentile on day 7 for the baseline dataset and day 28 for the follow-up dataset. We generated four datasets, including a baseline length dataset (89 non-EUGR infants and 35 EUGR infants), a baseline weight dataset (95 non-EUGR infants and 35 EUGR infants), a follow-up length dataset (41 non-EUGR infants and 83 EUGR infants), and a follow-up weight dataset (52 non-EUGR infants and 72 EUGR infants). In the baseline datasets, the birth weight and length of the non-EUGR group were higher than the EUGR group. Contrary to the length dataset in which the maternal age was greater in non-EUGR infants, the maternal age in baseline weight dataset was lower in the non-EUGR infants than the EUGR infants. Detailed baseline infant characteristics (weight and length) are presented in Table 1 and follow-up infant characteristics (weight and length) are presented in Table 2.

### 3.2 Correlation analysis

**Figure 2:**
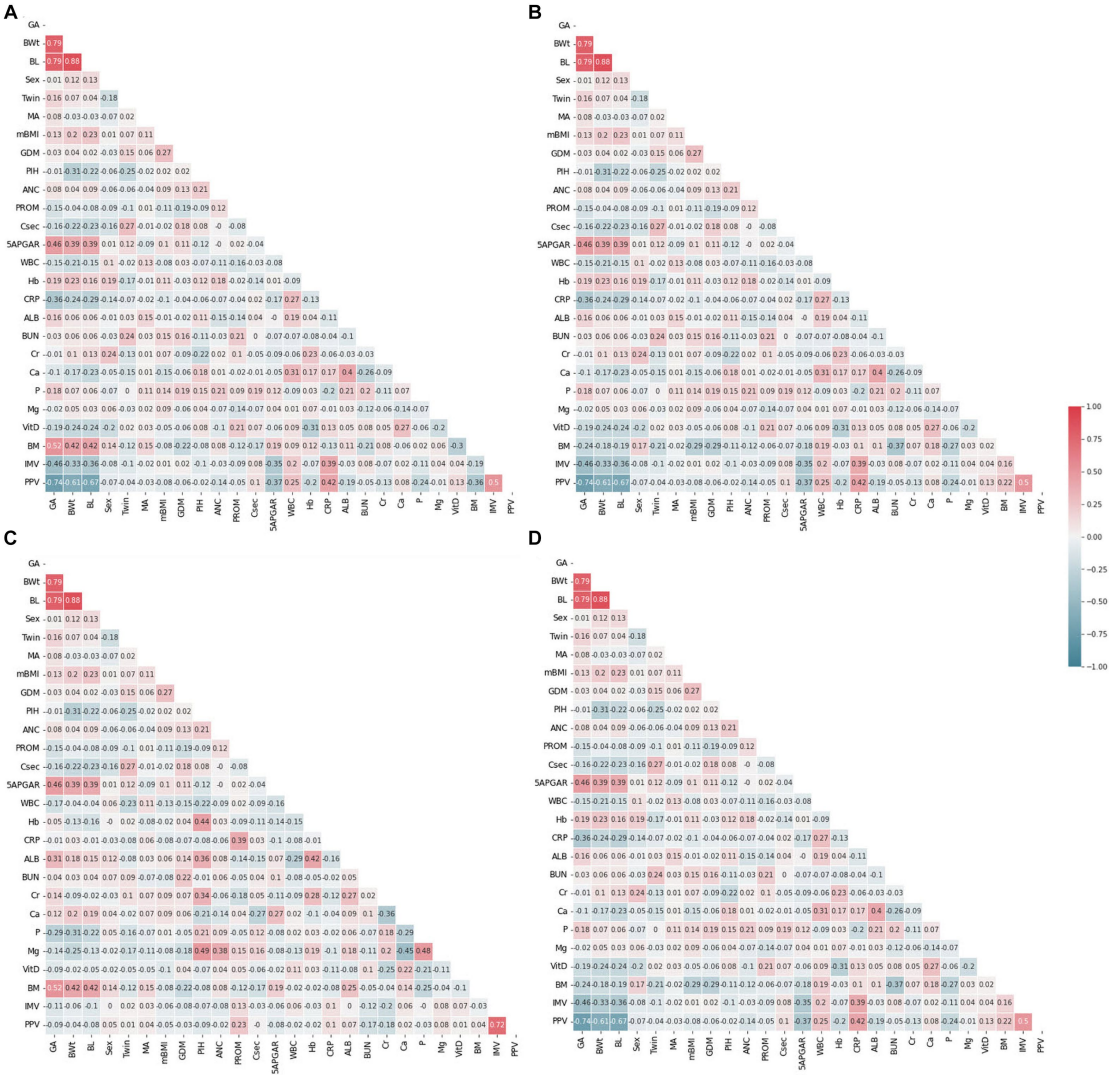
Feature correlation analysis with heatmaps. **(A)** Weight baseline; **(B)** weight follow-up; **(C)** length baseline; **(D)** length follow-up. Positive impact sizes are represented by hues of red, while negative effect sizes are represented by shades of blue.

Correlation is a statistical approach that determines a relationship between two or more variables with one another. The Pearson coefficient is an indicator used to measure the strength and direction of a linear relationship between given variables and responses (46). The heatmap generated by Pearson correlation has been commonly used in numerous research fields (47–49). The study conducted a correlation analysis to gain an initial understanding of the relationships between the predictor variables and the outcome variables in all datasets. By comparing the correlation coefficients in the baseline and follow-up datasets, the study aimed to identify potential trends and changes in the factors affecting the outcome variables (weight and length) in the context of EUGR. This analysis was a crucial step in exploring the structure of the datasets and selecting appropriate statistical models for subsequent analyses. The absence of evident multicollinearity was an important diagnostic finding, as it ensured that the assumptions of the selected models were met and that the results were valid and interpretable. The Pearson correlation coefficient, like other correlation measurements, can be positive or negative between −1 and + 1 in value. A positive correlation means that the variables increase or decrease together. A negative correlation suggests that if one variable increases, the other decreases, and vice versa. The correlations between predictors are shown as feature-correlation heat maps in Figure 2. Color type and intensity are used to indicate the degree of correlation. Detecting multicollinearity problems requires demonstrating a lack of strong correlation between the covariates (50, 51). To check for instances of multicollinearity problems, Pearson correlation coefficients were determined. As revealed by the heat maps in Figure 2, all four datasets were free of multicollinearity among the variables.

**Figure 3:**
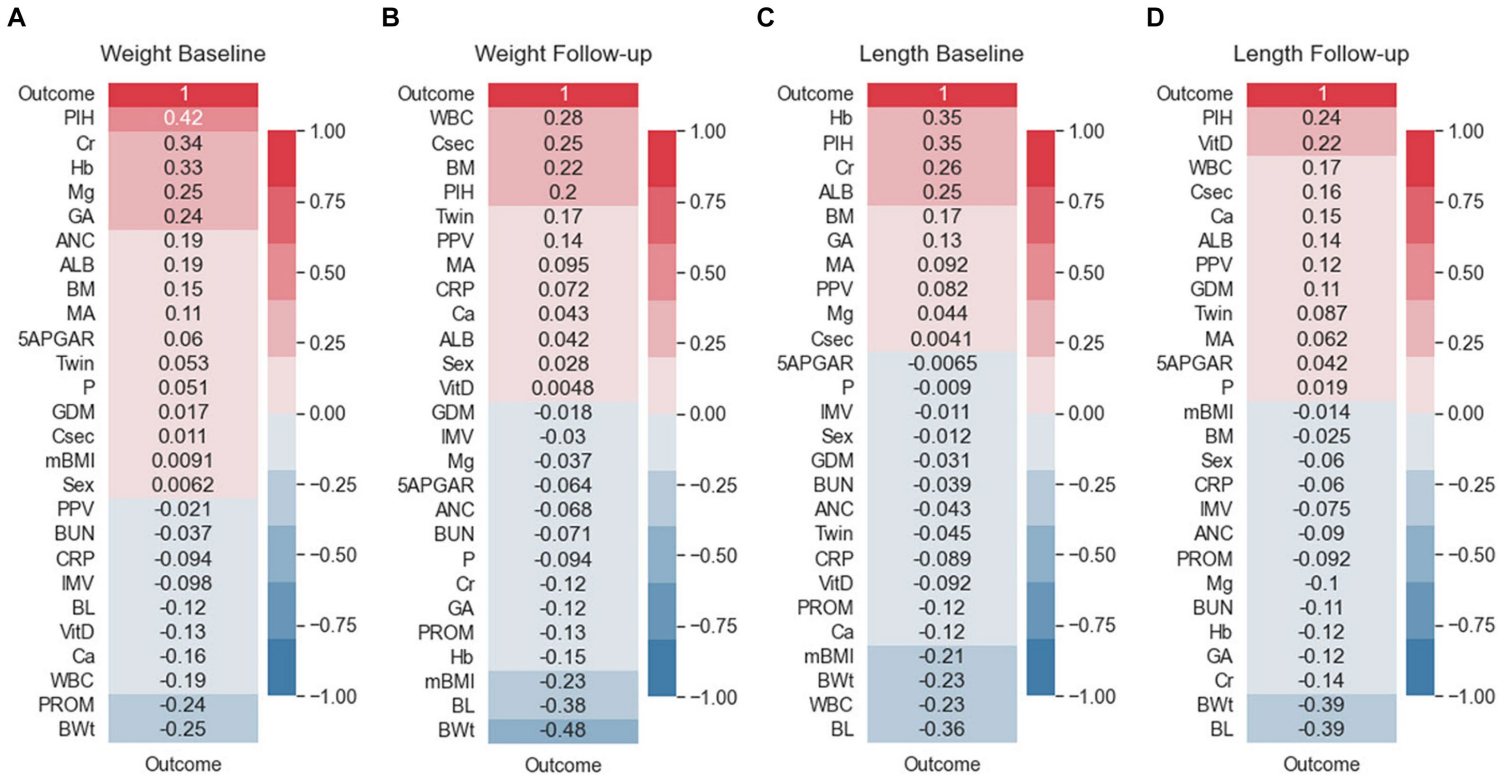
Feature correlation with the EUGR outcome. **(A)** Weight baseline; **(B)** weight follow-up; **(C)** length baseline; **(D)** length follow-up. Positive impact sizes are represented by hues of red, while negative effect sizes are represented by shades of blue.

The correlation between predictor variables and the target variable can be a significant indicator as the predictor variables that tend to have a high correlation with the target variable but exhibit low inter-correlation are efficient for classification tasks. To consider the existence of a correlation between predictor variables and the target variable, Pearson correlation coefficients were evaluated. Figure 3 shows the Pearson correlation coefficients between the target variables. As reflected in this figure, PIH exhibited the highest correlation with the target in the weight bassline and length follow-up groups. The follow-up birth length and birth weight showed high negative correlation with the target, and a strong positive correlation was found for different follow-up datasets. Among the top ten most positive correlations with the target, PIH, creatinine (Cr), Hemoglobin (Hb), gestational age (GA), albumin, maternal age MA, and breast milk (BM) were the same for the baseline datasets. However, considering the ten top negative correlations, birth weight (BWt), white blood cells count (WBC), PROM, birth length (BL), Vitamin D (Vit D), calcium (Ca), and C-reactive protein (CRP) were the same for follow-up datasets. Conversely, maternal body mass index (mBMI) in the weight baseline, Apgar score at 5 min (5APGAR) in the length baseline, Vit D in the weight baseline, and mBMI in the length follow-up datasets were demonstrated to exhibit the lowest correlation with the target.

### 3.3 Comparison of model performances

Table 3 presents the results achieved by the algorithms according to the selected performance metrics. When comparing the baseline and follow-up datasets in terms of accuracy, the baseline datasets for weight and length obtained a better performance than the follow-up datasets. Furthermore, when comparing the baseline datasets, the weight dataset achieved a better performance than the length dataset for both the baseline and follow-up datasets. Regarding the classifiers, our study found that the LR algorithm exhibited the best accuracy, with 83.07% for the weight baseline and 74.97% for the length baseline datasets, which demonstrates that the LR model performed better than the SVM model on the two baseline datasets. However, the SVM model performed better on the follow-up datasets. Our results indicate that weight-based classification, using either the baseline or follow-up approach, can provide a reliable benchmark for disease diagnosis. The better performance of SVM might be attributed to its geometric method that maximize the margins to each class, aiding in dividing the feature space with a more accurate decision boundary than LR. Overall, our study highlights the importance of selecting the appropriate algorithm for the dataset and considering the context in which the algorithm will be applied.

**Table 3:** Test results of all evaluated algorithms.

ML models	Accuracy (%)	Precision (%)	Recall (%)	F1-score (%)
**Weight baseline**				
LR	83.07 ± (0.04)	80.00 ± (0.24)	33.48 ± (0.11)	47.10 ± (0.15)
SVM	79.03 ± (0.05)	65.33 ± (0.30)	29.48 ± (0.11)	38.83 ± (0.14)
**Weight follow-up**				
LR	72.47 ± (0.12)	77.78 ± (0.14)	87.61 ± (0.14)	80.51 ± (0.09)
SVM	74.10 ± (0.09)	76.20 ± (0.13)	91.49 ± (0.07)	82.01 ± (0.06)
**Length baseline**				
LR	74.97 ± (0.09)	76.67 ± (0.37)	22.44 ± (0.16)	32.82 ± (0.21)
SVM	72.60 ± (0.07)	48.67 ± (0.33)	15.56 ± (0.02)	22.35 ± (0.13)
**Length follow-up**				
LR	65.50 ± (0.08)	68.01 ± (0.08)	77.22 ± (0.12)	71.40 ± (0.06)
SVM	66.90 ± (0.08)	68.30 ± (0.08)	84.21 ± (0.12)	74.60 ± (0.05)

AUC, area under the ROC curve; LR, logistic regression; SVM, support vector machine.

Precision and recall are important metrics in medical diagnosis because they measure the accuracy and completeness of a diagnostic test. They are widely used in medical research and are valuable tools for improving the accuracy and reliability of diagnostic tests (52–54). Precision measures how well a test identifies true positive cases, while recall measures how well it detects all positive cases, including true and false positives. Inaccurate results can have serious consequences for patients, making it essential to evaluate diagnostic tests using metrics that account for both the accuracy and completeness of the results. On the Baseline dataset, the LR model has better precision on both the weight and length data. The LR model scored 80.00 ± (0.24) on the weight baseline and 76.67 ± (0.37) on the length baseline, while the SVM model scored 65.33 ± (0.30) and 48.67 ± (0.33) respectively. This means that the LR model was better at avoiding false positives in both cases. Regarding recall score, the LR model also has better recall on the weight data with a score of 33.48 ± (0.11), compared to SVM’s 29.48 ± (0.11). However, in the length data, the LR model’s recall is only slightly better than SVM’s, with scores of 22.44 ± (0.16) and 15.56 ± (0.02) respectively. For F1-Score, the LR model has better F1-Scores on both the weight and length data, scoring 47.10 ± (0.15) and 32.82 ± (0.21) respectively, compared to SVM’s 38.83 ± (0.14) and 22.35 ± (0.13).

When comparing the follow-up dataset, the precision is quite close between the models. The LR model scored slightly higher than the SVM model on the weight data, 77.78 ± (0.14) vs. 76.20 ± (0.13), but slightly lower on the length data, 68.01 ± (0.08) versus 68.30 ± (0.08). In terms of recall, the SVM model has better performance on both the weight and length data. The SVM model’s recall scores were 91.49 ± (0.07) and 84.21 ± (0.12), while the LR models were 87.61 ± (0.14) and 77.22 ± (0.12) respectively. For the F1-Score, the SVM model scored higher on the weight data, 82.01 ± (0.06), compared to LR’s 80.51 ± (0.09). However, there seems to be an error in your report for the length data. The SVM’s F1-Score is reported as 4.60 ± (0.05), which is much lower than expected. The LR model’s F1-Score for the length data is 71.40 ± (0.06). In conclusion, on the baseline dataset, the LR model outperforms the SVM model in terms of precision, recall, and F1-Score for both the weight and length data. This suggests that the LR model is more reliable for predicting baseline conditions, with fewer false positives and false negatives. On the Follow-up dataset, the situation exhibits more complexity. For precision, the models are quite close, with the LR model doing slightly better on the weight data, and the SVM model doing slightly better on the length data. For recall, the SVM model performs better on both the weight and length data, suggesting that it might be better at catching positive cases in the follow-up data. However, the F1-Score, which balances precision and recall, is better for the LR model on the weight data and better for the SVM model on the length data, assuming there’s a mistake in the reported SVM F1-Score for the Length data.

**Figure 4:**
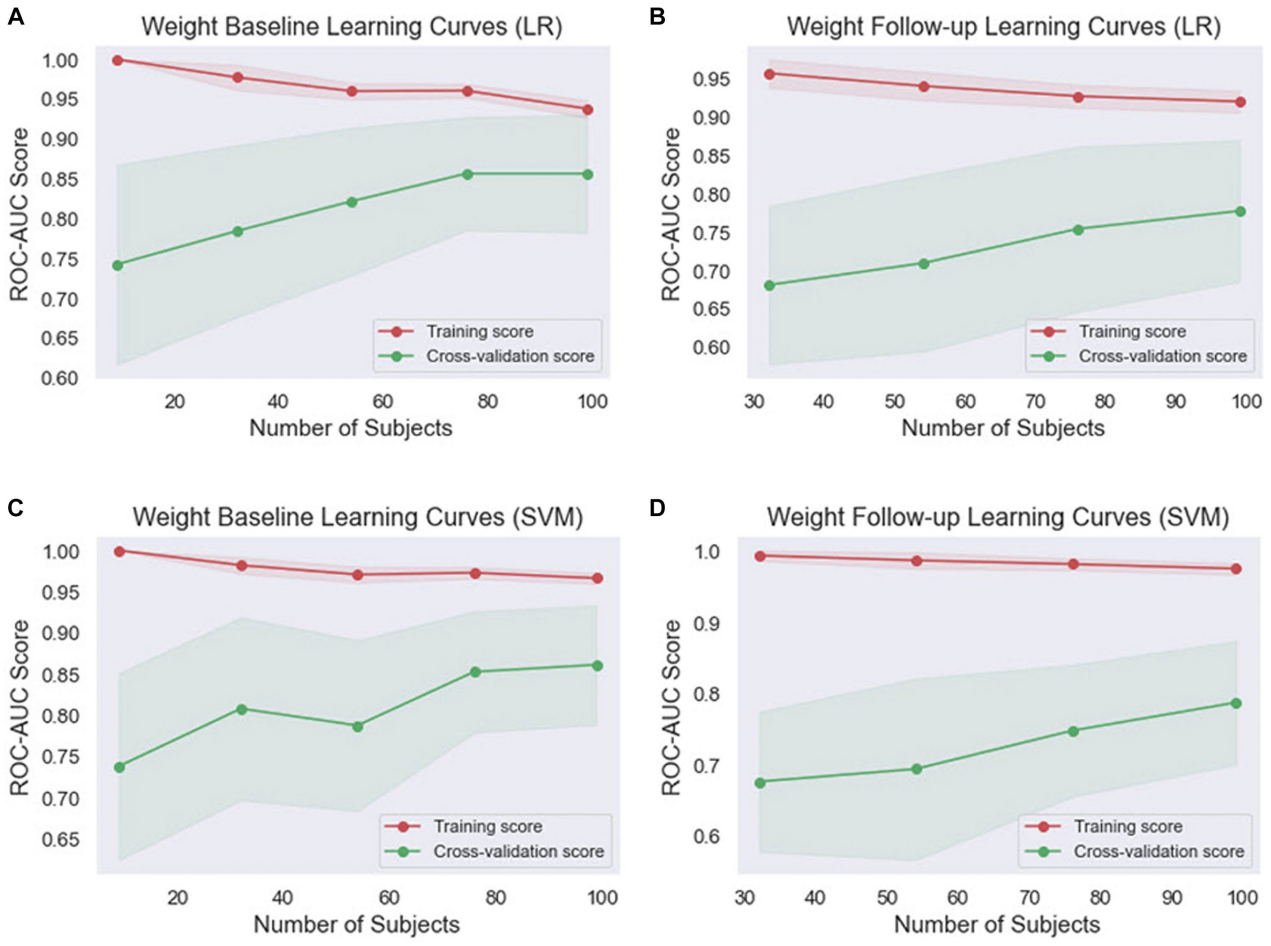
Comparison of the learning curves generated by the machine learning models for weight-based datasets based on the number of samples using five-fold cross-validation. **(A)** Weight baseline with LR; **(B)** weight follow-up with LR; **(C)** weight baseline with SVM; **(D)** weight follow-up with SVM. LR, logistic regression; SVM, support vector machines.

**Figure 5:**
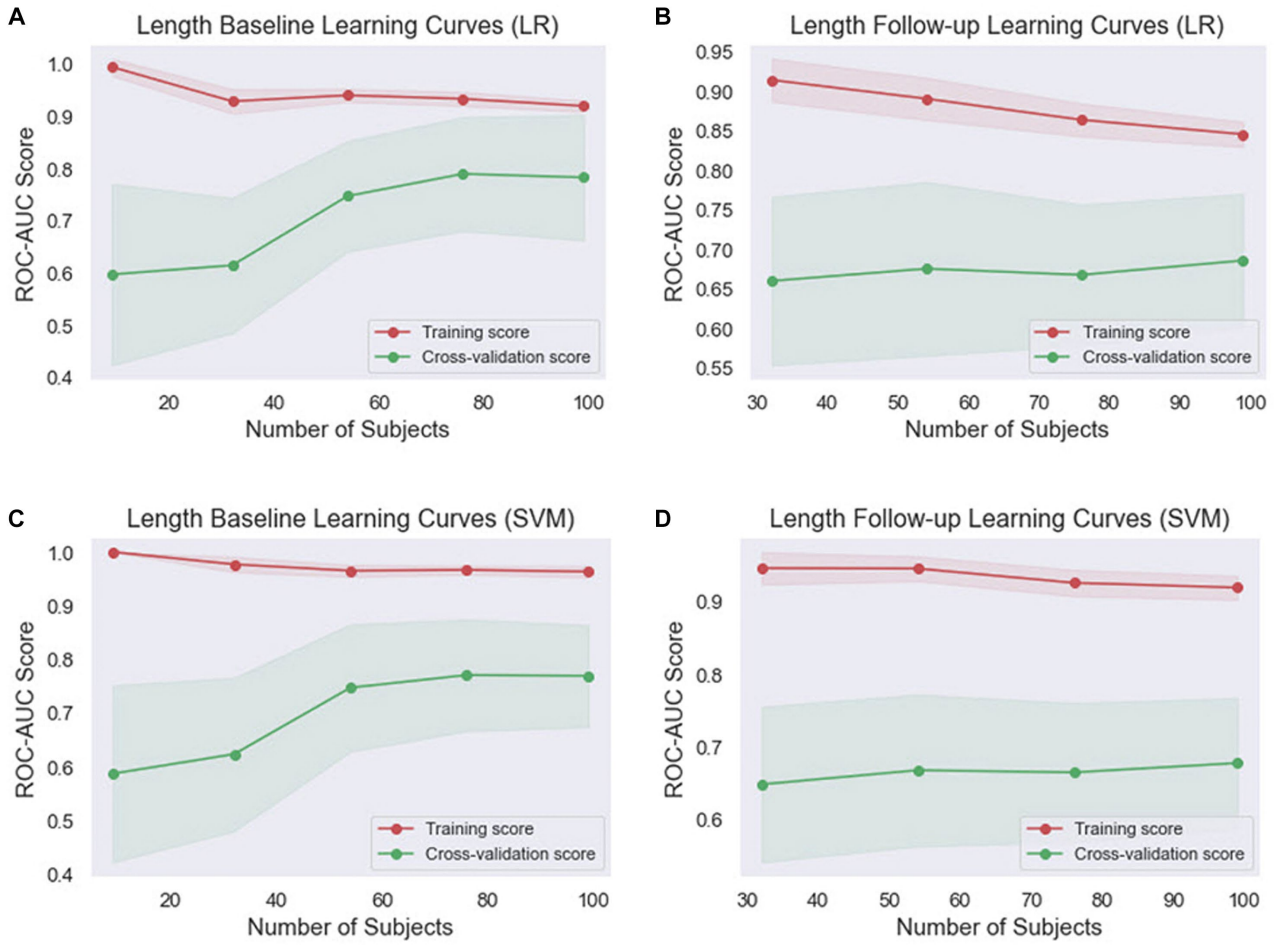
Comparison of the learning curves generated by the machine learning models for length-based datasets based on the number of samples using five-fold cross-validation. **(A)** Length baseline with LR; **(B)** length follow-up with LR; **(C)** length baseline with SVM; **(D)** length follow-up with SVM. LR, logistic regression; SVM, support vector machines.

Given that ML models tend to overfit to small datasets, especially in the medical field, it is important to investigate the occurrence of overfitting (55). It can also help determine if additional training examples could improve the model’s performance. The performance associated with repeated tasks improves with experience, practice, and training (56). This improvement is typically very quick at first but then gradually slows down (57). This process is sometimes referred to as a learning curve. In ML, a learning curve is a common diagnostic tool that shows how a model’s performance varies when more or less training samples are utilized (58). Learning curves can be used to determine if more training examples can improve validation scores. This measure also detects underfit, overfit, and well-fit models. In this study, we employed learning curves to evaluate the learning capacities of prediction models with varying amounts of training data (Figures 4, 5). In the weight baseline dataset (Figures 4A,C), both LR and SVM models demonstrate high training scores that gradually decrease as the number of samples increases, indicating that these models are complex enough to fit the data accurately. However, the cross-validation scores paint a different picture. The LR model’s score starts at 74 and increases to 85, indicating that it is improving its ability to generalize to unseen data. The SVM model starts at a similar score and slightly outperforms the LR model with a final score of 87, suggesting that it might be better at generalizing on this dataset. Similarly, in the weight follow-up dataset (Figures 4B,D), the LR model’s training score starts high and slightly decreases, while the cross-validation score increases, indicating effective learning. The SVM model’s training score decreases slightly more than the LR models, but its cross-validation score improves and ends slightly higher than the LR model’s.

Concerning the length baseline data (Figures 5A,C), the LR model’s training score starts high and decreases more significantly, while the cross-validation score increases from 60 to 81, indicating effective learning. The SVM model’s training score decreases slightly, while its cross-validation score also rises significantly but ends slightly lower than the LR model’s. For the length follow-up data (Figures 5B,D), both models’ training scores decrease, but the SVM model’s decreases less. Their cross-validation scores also rise, but less dramatically than for the baseline data.

Overall, the SVM model generally maintains higher training scores, suggesting that it might be better at fitting the data or potentially overfitting. However, its cross-validation scores are generally comparable to or slightly higher than the LR model’s, indicating that it might be slightly better at generalizing. These findings indicate that both models are learning effectively from the data and are not suffering from underfitting or overfitting. The choice between the two models may depend on the specific requirements of the task, such as the importance of precision or recall.

**Figure 6:**
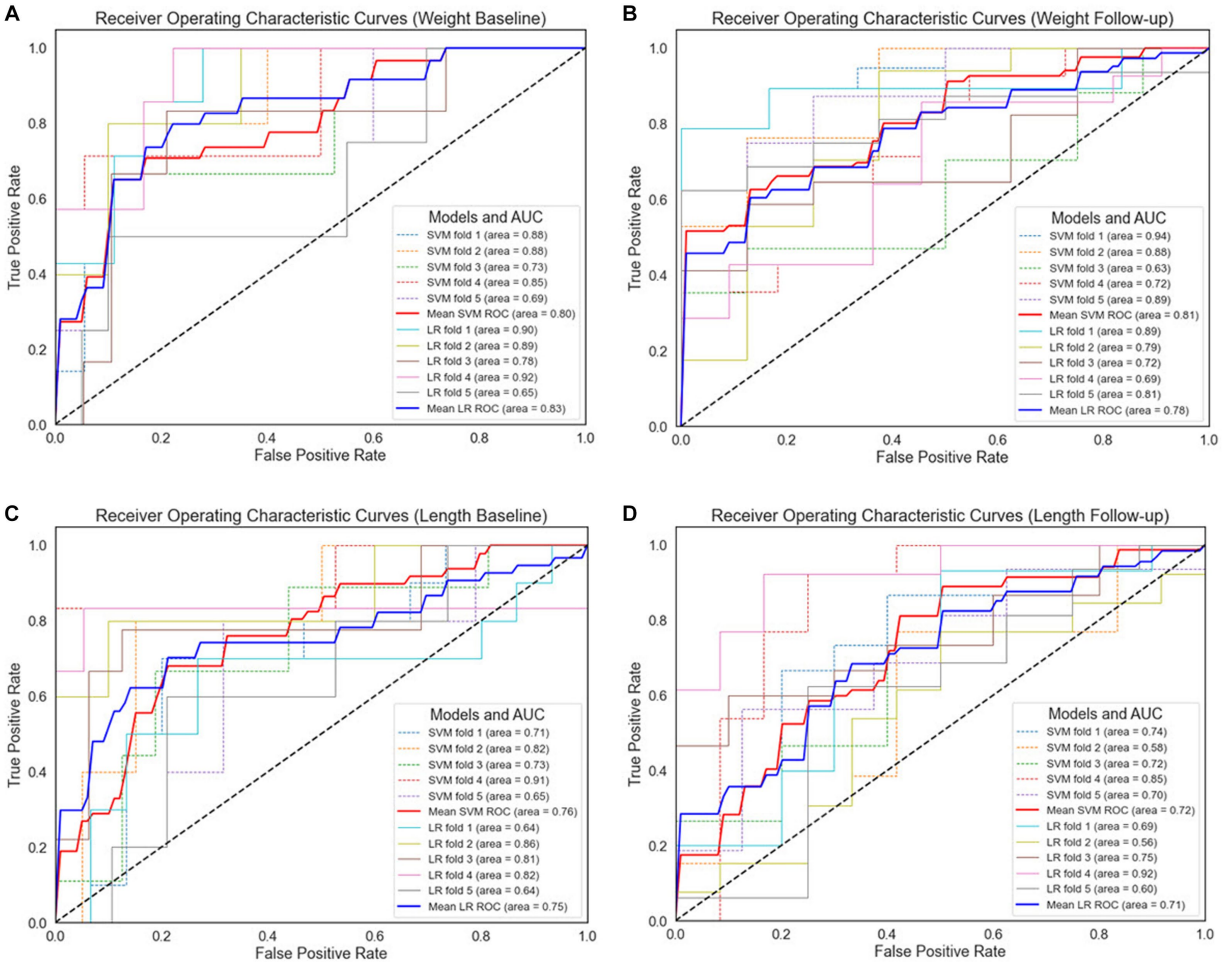
Receiver operating characteristic curves for 5-fold cross-validation. **(A)** Weight baseline; **(B)** weight follow-up; **(C)** length baseline; **(D)** length follow-up. AUC, area under the receiver operating characteristic curve; ROC, receiver operating characteristic curves; LR, logistic regression; SVM, support vector machines.

**Figure 7:**
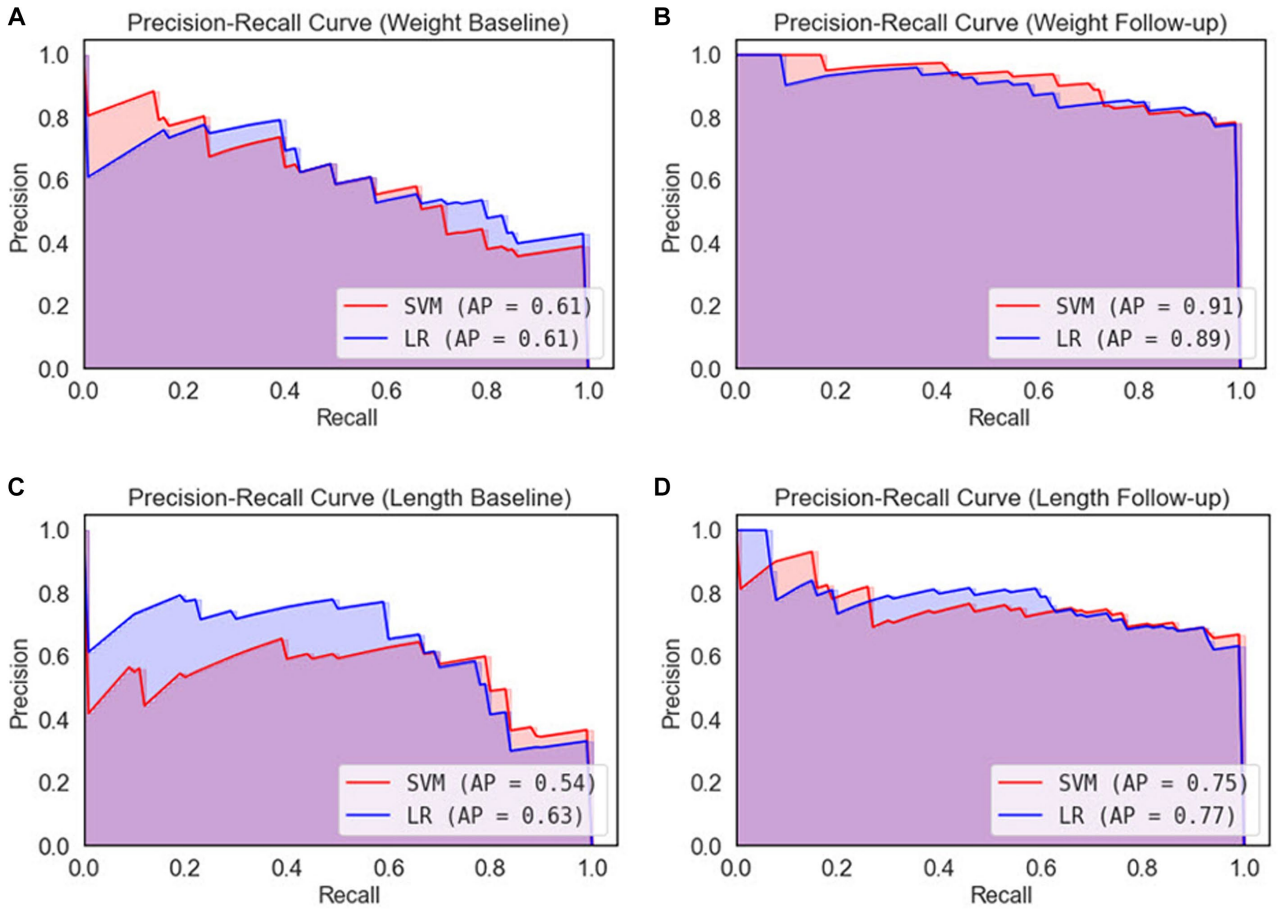
Precision-recall curves for 5-fold cross-validation. **(A)** Weight baseline; **(B)** weight follow-up; **(C)** length baseline; **(D)** length follow-up. AP, average precision; LR, logistic regression; SVM, support vector machines.

The models were further evaluated using the ROC curve and precision–recall curve (PRC) parameters. ROC shows the relationship between the rate of true positives and false positives, while precision–recall (PR) curve and the area under it is widely used to summarize the performance of machine learning classifier results, especially when evaluating classifiers on imbalanced datasets. This curve represents the tradeoff between the proportion of positively labeled examples that are truly positive (precision) as a function of the ratio of correctly classified positives (recall). The ROC and PRC curves for the different types of EUGR ML classification are presented in Figures 6, 7 respectively.

For the weight-based dataset, we observe that both models demonstrate comparable performance, as indicated by their respective AUC values. Specifically, the SVM model achieved an AUC of 80% with the baseline data (Figure 6A), while LR outperformed slightly with an AUC of 83%. In Figure 5B, the SVM model improved slightly to 81%, surpassing the performance of the LR model in weight follow-up dataset which displayed a slight decrease to 78%. This suggests that the SVM model may perform better under certain conditions or with certain data subsets within the weight-based dataset. However, given the marginal difference in performance, both models can be considered competent for this dataset.

Regarding the length-based dataset, the performances of the two models were more closely matched. In Figure 5C, the SVM model achieved an AUC of 76%, marginally outperforming the LR model, which achieved an AUC of 75%. Similar performance was observed in length follow-up dataset as demonstrated in Figure 5D, where the SVM model and the LR model achieved AUCs of 72 and 71%, respectively. While the SVM model displayed slightly superior performance in both cases, the difference is minor, suggesting that both models are similarly effective when applied to the length-based dataset.

Therefore, it can be concluded that SVM outperformed LR in all comparison results. Comparing the baseline datasets, the ROC value of the weight dataset is higher than the length dataset. This trend was also observed for the follow-up datasets, in which weight-based datasets performed better than length-based datasets. When comparing between baseline and follow-up generated datasets, the latter performs better. In contrast, in length format, length baseline performs better than length follow-up.

After performing ROC analysis, we next investigated the Precision-Recall Curve (PRC) to evaluate the performance of our ML models. The results are illustrated in Figure 7. When examine the models’ performance on the weight dataset, as illustrated in Figure 7A, both the SVM and LR models have identical PR scores of 61%. However, in weight follow-up (Figure 7B), we notice a substantial improvement in performance, with the SVM model attaining a PR score of 91%, slightly outperforming the LR model that scored 89%. On the basis of the weight dataset, it is clear that the SVM model displays marginally superior performance. Comparing the length dataset, as it is demonstrated in Figure 7C the LR model outperformed the SVM model, achieving a PR score of 63% compared to the SVM’s 54% in length baseline. Similarly, in Figure 7D, the LR model again demonstrated better performance with a PR score of 77%, slightly higher than the SVM model’s score of 75%. From the perspective of the length dataset, it is evident that the LR model exhibits better performance than the SVM model.

Based on the performance of both models across all datasets, it appears that the SVM model shows a slightly better performance on the weight dataset, while the LR model is more proficient on the length dataset. However, the disparity in PR scores across the models is relatively small, suggesting that both models demonstrate comparable performance.

**Figure 8:**
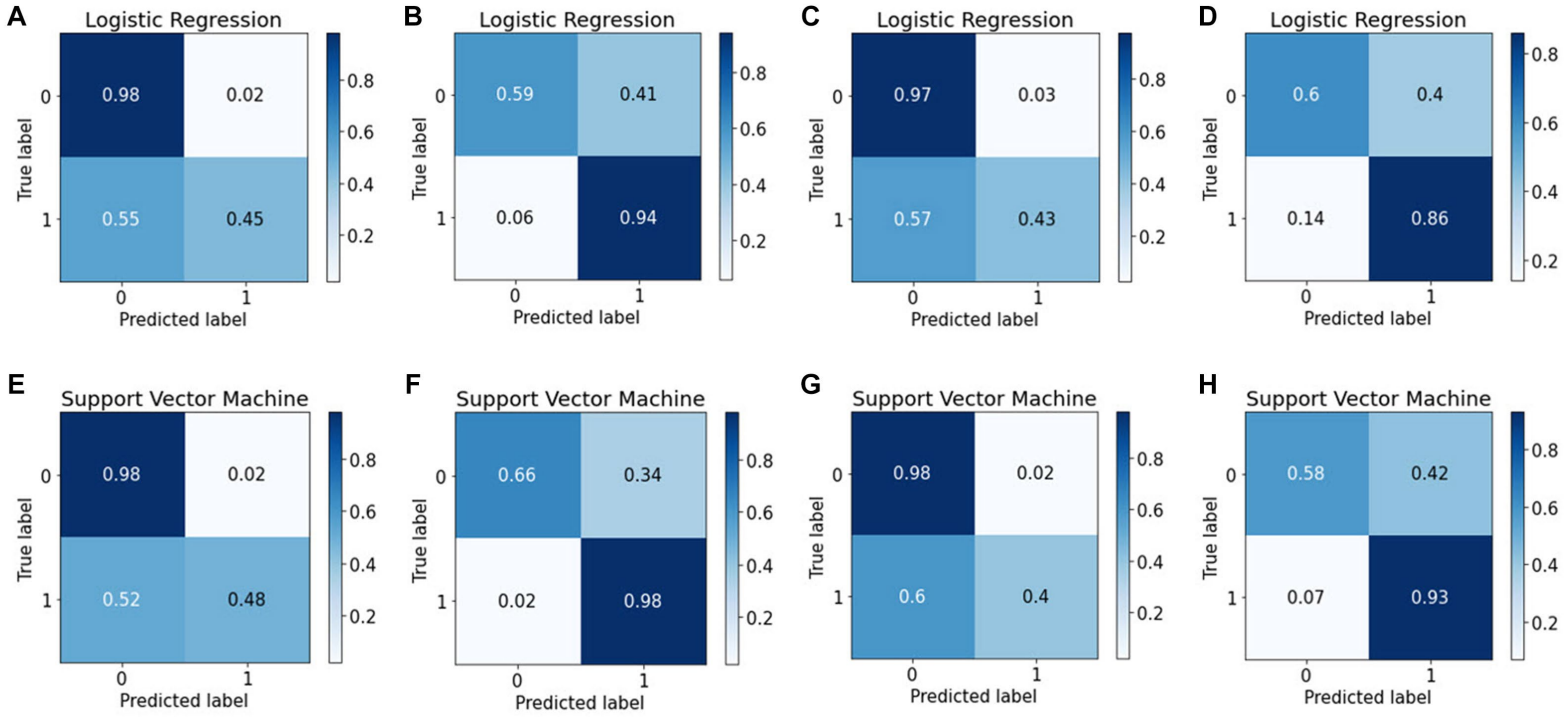
Confusion matrices of the logistic regression and support vector machine models. **(A)** Weight baseline; **(B)** weight follow-up; **(C)** length baseline; **(D)** length follow-up; **(E)** weight baseline; **(F)** weight follow-up; **(G)** length baseline; **(H)** length follow-up with.

The confusion matrix is more extensively applied than classification accuracy because it provides a clearer overview of a model’s performance. Consider classification accuracy; there is currently no way to learn the percentage of incorrect labels. Conversely, the confusion matrix will provide more insight into a classifier’s performance because it shows the correctly and incorrectly classified cases for all classes. To evaluate the performance of the ML algorithms in classifying EUGR, the confusion matrix for the binary classification tasks were calculated to obtain disease-wise classification performance of the models. The rows represent the actual class, whereas the columns represent the predicted class. The confusion matrix obtained by the machine learning models for EUGR and non-EUGR classification is shown in Figure 8. In the weight dataset, the LR model demonstrated a non-EUGR accuracy of 98% and an EUGR accuracy of 45% at baseline (Figure 8A). At the follow-up stage, the non-EUGR accuracy decreased to 59%, whereas the EUGR accuracy significantly increased to 94% (Figure 8B). On the other hand, the SVM model showed similar non-EUGR accuracy at baseline (98%) but slightly higher EUGR accuracy (48%) (Figure 8E). In the follow-up stage, the non-EUGR accuracy slightly increased to 66% compared to the LR model, and the EUGR accuracy reached an impressive 98% (Figure 8F). Thus, based on the weight dataset, the SVM model outperformed the LR model, particularly in terms of EUGR accuracy in the follow-up stage.

Turning to the length dataset, the LR model achieved a non-EUGR accuracy of 97% and an EUGR accuracy of 43% at baseline (Figure 8C), with the follow-up stage showing a decrease in non-EUGR accuracy to 60% but an increase in EUGR accuracy to 86% (Figure 8D). In comparison, the SVM model displayed a higher non-EUGR accuracy of 98% and a slightly lower EUGR accuracy of 40% at baseline (Figure 8G). The follow-up stage revealed a decrease in non-EUGR accuracy to 58% but an increase in EUGR accuracy to 93% (Figure 8H). Considering these results, the SVM model’s performance is comparable to the LR model’s performance when using the length dataset, with a slight edge in EUGR accuracy in the follow-up stage.

Comparing both models across all datasets, the SVM model appears to have a slight edge over the LR model. While both models show similar non-EUGR accuracy at baseline, the SVM model consistently displays higher EUGR accuracy in the follow-up stage, regardless of the dataset used. This suggests that the SVM model may be better at capturing the complexity of the data and providing accurate predictions over time. However, the decision between using SVM or LR should consider the specific requirements of the task, such as the relative importance of non-EUGR and EUGR accuracy and the potential changes in these metrics over time.

## 4 Model trustworthy and interpretability

**Figure 9:**
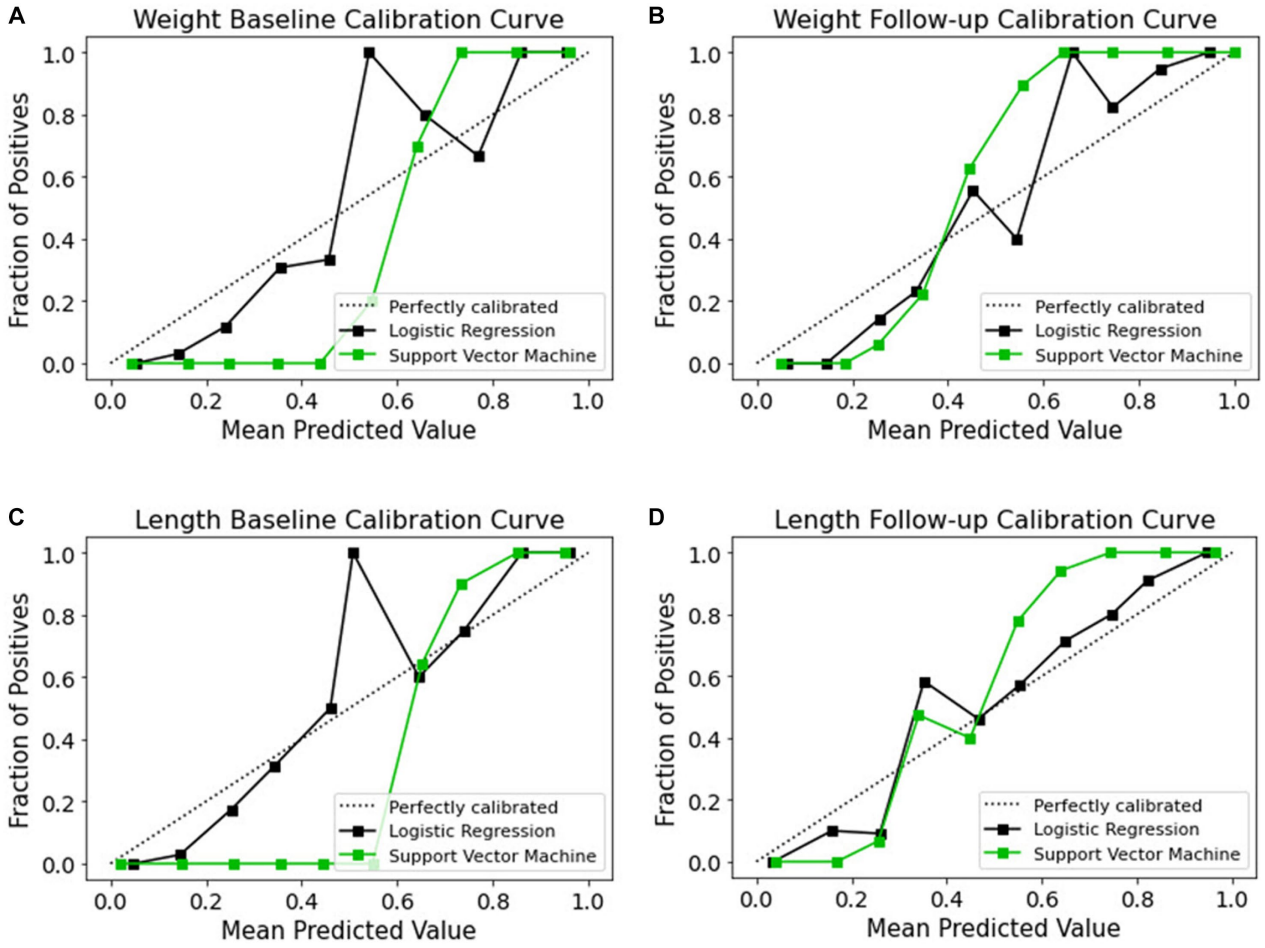
Calibration curves of models for the four datasets. **(A)** Weight baseline; **(B)** weight follow-up; **(C)** length baseline; **(D)** length follow-up. The dotted lines indicate the optimal probability prediction model, while the solid line represents the obtained data.

Although ML-based models hold potential for clinical adoption, their trustworthiness and interpretability are often disregarded. Recent years have seen an increasing tendency toward making ML models more open, with an emphasis on revealing the inner working of black-box algorithms via post-hoc, model-agnostic techniques to help the user grasp the model’s working (59–61). Accurate probabilistic predictions are crucial in medicine to ensure trustworthiness. However, the process of model calibration and the learning of well-calibrated probabilistic models have not been explored as thoroughly as discriminative ML models, which are designed to maximize class discrimination. Evaluating the calibration process is a crucial step in developing and verifying clinical prediction models. Therefore, it is essential to investigate the calibration of probabilistic models, especially in medical applications, to ensure reliable predictions and improve the overall quality of clinical decision-making. The term “calibration” refers to the degree to which the expected risk aligns with the actual risk (62). The calibration curve shows the linear relationship between the independent and dependent (response) variables using the least-squares method (63). The data are categorized into groups that are referred to as bins. The probability predicted by a classifier is shown along the *x*-axis, while the number of positive examples found in each bin is shown along the *y*-axis. The closer the generated calibration curves are to the standard line, the more the model’s predictions align with the actual class distribution in the dataset. In recent decades, the assessment of the calibration performance of risk prediction models based on ML algorithms have received considerable attention in the medical field (64, 65). In a classification task, a calibration curve plot can demonstrate the comparison of two machine learning models in terms of their calibration performance. A calibration curve shows the relationship between the predicted probability and the true probability of the positive class for a binary classification task. The calibration performances of the prediction methods are illustrated in Figure 9. The calibration slope generated with the LR model from all datasets fit well with the optimal curves compared to those of the SVM model. Calibration curves for EUGR status predictions in the follow-up datasets (Figures 9B,D) demonstrated favorable performance than baseline datasets (Figures 9A,C). The calibrated LR classifier exhibited a good performance, as data were generated according to the dotted line, and outperformed the SVM classifiers.

**Figure 10:**
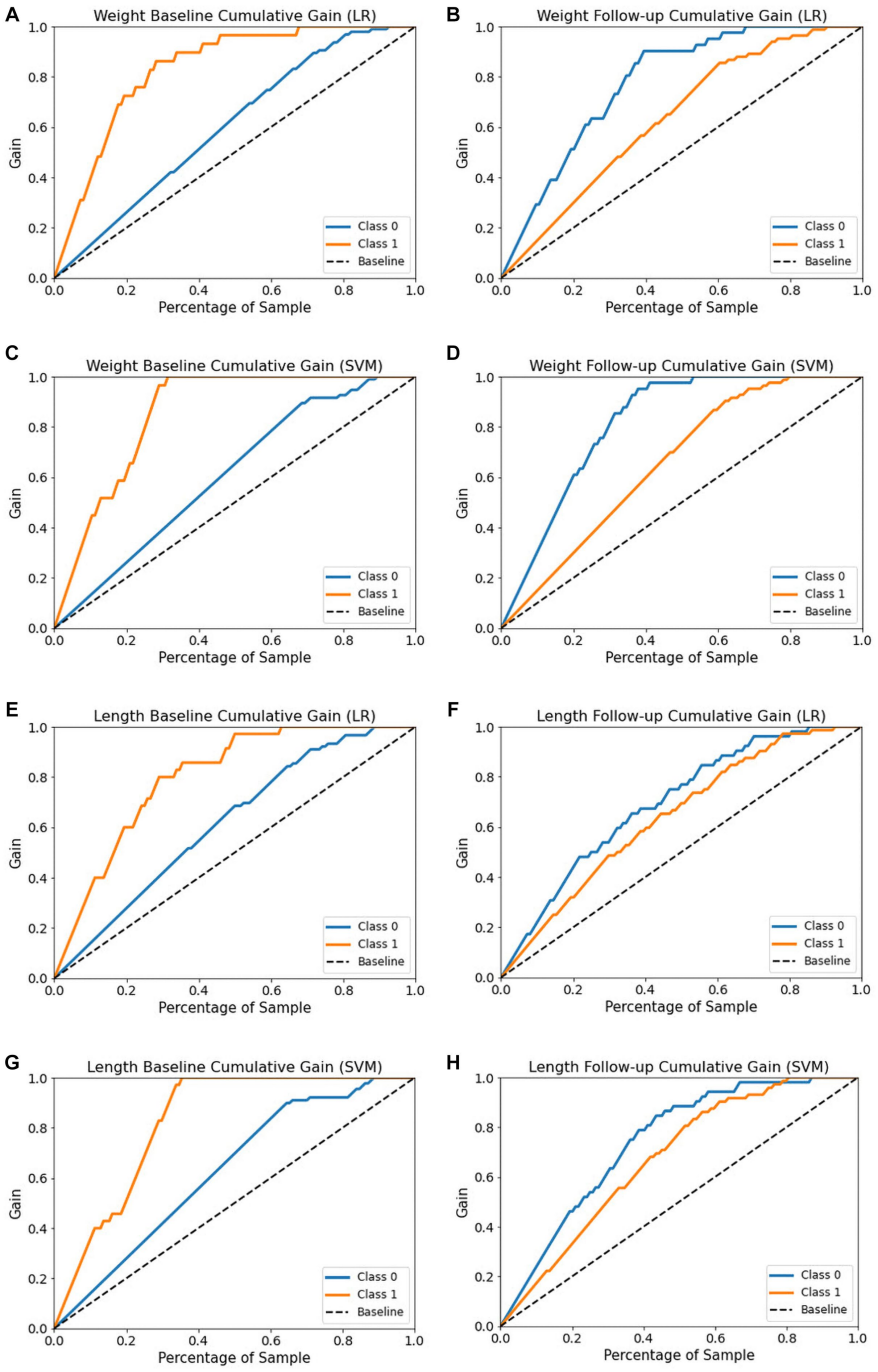
Cumulative gain curves of logistic regression and support vector machine models for the four datasets. **(A,C)** Weight baseline; **(B,D)** weight follow-up; **(E,G)** length baseline; **(F,H)** length follow-up. Class 0: non-EUGR; class 1: EUGR. LR, logistic regression; SVM, support vector machines.

The cumulative gains curve is a widely used visualization method that examines the performance and trustworthiness of a model and compares the outcome with a random selection (66, 67). It displays the proportion of positive targets achieved by considering a specific percentage of the population most likely to be positive, as per the model’s predictions. We also evaluated models’ trustworthy and reliability using cumulative gains as shown in Figure 10. In examining the weight baseline dataset, the LR and SVM models demonstrate better predictive potential. Upon screening 20% of the population, approximately 70 and 60% of EUGR patients could be identified using the LR and SVM models, respectively (Figures 10A,C). However, similar to the length dataset, the performance drops in the weight follow-up stage where both models could identify nearly 30% of actual high-risk patients within the top 20% of the population (Figures 10B,D).

With respect to the length baseline dataset, the cumulative gains curve reveals that selecting the top 20% of the population, considered high-risk for EUGR based on the LR model’s predictions, would contain approximately 60% of actual high-risk EUGR cases (Figure 10E). In contrast, SVM model predictions within the same population proportion would capture only 45% of the actual high-risk cases (Figure 10G). Nevertheless, when applied to the length follow-up dataset, both models show a substantial decrease, identifying only 30% of actual high-risk cases within the top 20% of the population (Figures 10F,H). In summary, these results emphasize the dynamic nature of model performance, highlighting the importance of continuous model evaluation and adjustment in predictive healthcare.

While current ML-based classification systems yield good prediction accuracy, a significant hurdle to their broad application is the lack of attention given by researchers to the problem of model interpretability (61, 68). In addition, considerable work is required to address the question of how effectively models can be perceived by humans. To improve the interpretation of ML methods, this study used the state-of-the-art Shapley additive explanation (SHAP) technique (41), to achieve global interpretability of applied models. This will allow to better understand the overall impact of each predictor variable on the EUGR target outcome. The Shapley value is borrowed from the field of game theory (69) and serves as the foundation for the SHAP approach. Table 4 ranks the contributions of all features based on their ranking for all datasets; the features are ordered in terms of their importance.

**Table 4:** Model interpretation using feature importance based on SHAP ranking.

Baseline				Follow-up			
Weight		Length		Weight		Length	
LR	SVM	LR	SVM	LR	SVM	LR	SVM
PIH	PIH	PIH	PIH	Twin	Csec	BWt	Twin
GA	ANC	BL	Twin	BWt	Twin	PIH	PIH
Twin	GA	GA	BL	PIH	BWt	BL	BWt
BWt	PPV	ANC	BWt	BM	BM	ANC	BL
ANC	BWt	BWt	GA	Csec	Sex	Twin	ANC
PROM	PROM	BM	PROM	Sex	PIH	GDM	Sex
Sex	Twin	mBMI	BM	BL	BL	5APGAR	GDM
PPV	GDM	PPV	GDM	mBMI	ANC	IMV	Csec
BL	Sex	PROM	PPV	GA	PROM	VitD	PROM
BM	5APGAR	Twin	mBMI	PROM	GDM	BUN	PPV
Csec	VitD	Csec	ANC	MA	mBMI	Csec	VitD
MA	BL	P	P	WBC	WBC	PROM	5APAGAR
P	ALB	Ca	BUN	ANC	PPV	WBC	BUN
5APGAR	BM	Sex	Sex	IMV	GA	BM	MA
BUN	BUN	BUN	Mg	P	MA	ALB	ALB
WBC	Mg	MA	VitD	Hb	Hb	GA	IMV
Ca	MA	ALB	Cr	VitD	VitD	Cr	BM
VitD	WBC	VitD	Ca	Cr	ALB	MA	GA
GDM	P	Mg	MA	5APGAR	P	Ca	Hb
IMV	Cr	5APGAR	IMV	Ca	5APGAR	Hb	WBC
ALB	Ca	GDN	WBC	GDM	BUN	Sex	mBMI
Hb	mBMI	IMV	Csec	ALB	Ca	CRP	P
Mg	Hb	Hb	5APGAR	CRP	Cr	Mg	Ca
Cr	IMV	Cr	Hb	Mg	CRP	mBMI	CRP
CRP	CRP	WBC	ALB	BUN	Mg	P	Cr
mBMI	Csec	CRP	CRP	PPV	IMV	PPV	Mg

BL, birth length; BWt, birth weight; GA, gestational age; BMI, body mass index; GDM, gestational diabetes mellitus; PIH, pregnancy-induced hypertension; ANC, antenatal corticosteroid; PROM, premature rupture of membrane; Csec, Cesarean section; 5APGAR, Apgar score at 5 min.; WBC, white blood cells; Hb, hemoglobin; CRP, C-reactive protein; BUN, blood urea nitrogen; Cr, creatinine; Ca, calcium; P, phosphorous; Mg, magnesium; Vit D, vitamin D; MV, mechanical ventilation; PPV, positive pressure ventilation.

As reflected in Table 4, in weight baseline dataset for both LR and SVM models, PIH, GA, Twin, and BWt are considered the most influential features. However, the LR model ranks ANC higher than the SVM model, whereas the SVM model ranks PPV higher than the LR model. On the lower end, the LR model considers mBMI, CRP, and Mg as less impactful, while the SVM model finds Csec, CRP, and mBMI to be less influential. In weight follow-up dataset, the LR model places high importance on Twin, BWt, PIH, and BM, while the SVM model ranks Csec, Twin, and BWt as top features. For weaker contributors, the LR model finds PPV as less impactful, while the SVM model ranks IMV, Mg, and CRP lower.

Regarding to length baseline dataset, both models rank PIH highly. The LR model emphasizes BL, GA, and ANC, while the SVM model considers Twin, BL, and BWt as top influencers. On the weaker side, the LR model finds PPV to be less impactful, whereas the SVM model ranks ALB, CRP, and Hb lower. In length follow-up dataset, for both models, BWt and PIH remain crucial, with the LR model also highlighting BL and ANC. The SVM model places more importance on Twin and BL. In terms of less influential features, the LR model ranks PPV lower, while the SVM model finds Cr, Mg, and P to be less impactful. In summary, the importance of features varies between the models and datasets. These insights can guide future feature selection and model refinement for EUGR prediction (Supplementary Figures S1, S2 for weight and length baseline dataset; Supplementary Figures S3, S4 for weight follow-up and length follow-up datasets).

## 5 Discussion

The use of AI models in the diagnosis of EUGR is gradually increasing. This study aimed to develop an outcome prediction model for infants using an explainable ML approach. We successfully generated a prediction model for preterm infants with EUGR using data sourced from the electronic health records at CHA Bundang Medical Center in South Korea. Four datasets were generated based on the weight and length (weight baseline, weight follow-up, length baseline and length follow-up) and we evaluated their effectiveness for predicting EUGR outcomes using 27 variables based on clinical and laboratory factors. These variables are the most clinically common and readily available, thereby further proving the model’s reliability can be used successfully when the baseline and follow-up datasets are varied.

Correlated variables are prevalent in high-dimension data. The correlation analysis was performed to provide initial insights into the relationships between the predictor variables and the outcome variables (weight-based and length-based) across all datasets (baseline and follow-up). The correlation analysis enables a comparison between the baseline and follow-up datasets, allowing us to observe how the relationships between the predictor variables and outcomes change over time and ensured the reliability of our model. We investigated the potential correlations between predictor variables and the target outcome. Our analysis revealed no significant collinearity within our dataset, suggesting that the correlations between the variables were mostly in line with expectations (Figure 2). PIH exhibited the most substantial correlation with the target outcome. Other variables, such as Cr, Hb, GA, ALB, MA, and BMR, were among the top 10 most positively correlated with the target in the baseline datasets. Conversely, when we examined the ten strongest negative correlations, we found that BWt, WBC, PROM, BL, VitD, Ca, and CRP were consistently featured across the follow-up datasets (Figure 3). In the medical diagnosis, reliable and accurate predictions are crucial, making the choice of machine learning model and its performance a critical aspect of any investigation. As presented in Table 3, we compared the performance of two widely used algorithms across four distinct datasets. When analyzing the baseline datasets for both weight and length, LR outperformed SVM in terms of accuracy, achieving 83.07 and 74.97%, respectively. This suggests that when dealing with initial baseline data, LR may provide more reliable predictions. However, this trend reversed in the follow-up datasets, where SVM demonstrated superior performance, indicating its potential for more accurate predictions in a time-series or sequential data context. Furthermore, our results emphasize that the nature of the data itself, whether it is weight or length, can also influence the model’s performance. For both the baseline and follow-up datasets, weight-based classification models generally outperformed length-based models for the prediction and classification of EUGR (Table 3). Our results suggest that weight-based classification, using either the baseline or follow-up approach, can serve as a reliable outcome indicator for disease diagnosis. This improved performance of weight-based datasets implies that using weight as the primary outcome variable may lead to more accurate and reliable classification of EUGR. It is important to note that our findings do not necessarily establish weight as the definitive benchmark for disease diagnosis. Instead, the results suggest that, in the context of our study and the datasets we analyzed, weight-based classification models performed better in identifying EUGR.

Precision and recall, key metrics in medical diagnosis, also played a significant role in evaluating model performance. These metrics provide a view of the model’s capability to identify true positive cases (precision) and detect all positive cases, including true and false positives (recall). In the baseline datasets, LR demonstrated superior precision and recall for both weight and length, suggesting its ability to minimize false positives and false negatives. However, in the follow-up datasets, the situation was more complex, with the SVM model demonstrating better recall in both weight and length data, implying its potential strength in identifying more true positive cases over time (Table 3). Additionally, we evaluated the F1-Score, a harmonic means of precision and recall, providing a balanced measure of the model’s performance. While LR outperformed SVM in the baseline datasets, the trend was not as clear in the follow-up datasets. We observed higher F1-scores for SVM in weight data, but the reported F1-Score for SVM in length data seemed unusually low, possibly indicating a reporting error.

Learning curves serve as valuable tools to measure a model’s performance during the training process, assessing whether the model is underfitting or overfitting the data. In our study, we observed the performance of LR and SVM models across two datasets, weight (Figure 4) and length (Figure 5). The training scores of both models for all datasets demonstrated a gradual decrease as the number of samples increased, indicating the complexity and adaptability of these models to fit the data accurately. While both models showed effective learning without signs of underfitting or overfitting, the SVM model consistently exhibited a slight advantage in generalizing from the training data. However, its cross-validation scores were typically comparable to or slightly higher than the LR model’s, suggesting it might be slightly better at generalizing.

Examining the ROC curve analysis, the SVM model displayed marginally superior performance, particularly with the follow-up data. However, the difference was minor, suggesting both models are effectively applicable to this dataset. Weight-based datasets performing better than length-based ones. Follow-up datasets generally outperformed baseline datasets, except in length where the baseline was superior. Precision-Recall curve analysis revealed that, in the context of the weight dataset, the SVM model displayed slightly superior performance, particularly with the follow-up data. In contrast, for the length dataset, the LR model outperformed the SVM model. Analyzing the confusion matrices (Figure 8) both models demonstrate high non-EUGR accuracy at baseline, but SVM consistently shows higher EUGR accuracy during follow-up, indicating its superior ability to capture the complexity of changes over time. This suggests that SVM may be more adept at handling complex, time-dependent clinical data.

When comparing with other related studies, Han et al. (31) achieved an AUROC of 74%, Leigh et al. (32) achieved a receiver operating characteristics performance of 92.10%, Wu et al. (33) achieved an AUROC of 88.10%, and Podda et al. (34) achieved an accuracy of 91.49%, our study achieved an AUC of 83 and 78% for the weight-based dataset using LR and SVM, respectively. For the length-based dataset, our study achieved an AUC of 75 and 71% using LR and SVM, respectively. While these results show comparable or slightly lower AUC performance than the related studies. However, it is important to consider the specific tasks and datasets used in each study, which can affect the performance of the machine learning models. Additionally, our study may have additional strengths in terms of the use of calibration plots, cumulative curves, and global interpretability, as well as correlation analysis. The use of calibration plots and cumulative curves can help evaluate the calibration and discrimination performance of a machine learning model, respectively, and provide insights into the model’s strengths and weaknesses. Similarly, global interpretability and correlation analysis can help identify the most important features and relationships between them, providing a better understanding of the underlying mechanisms driving the model’s predictions. These additional analyses can add value to the overall evaluation of the machine learning models and provide a more comprehensive assessment of their performance and potential clinical applicability.

Trustworthy ML has gained considerable interest in recent years, with the development of model explainability (70). This work aimed to enhance the interpretability of ML models by utilizing post-hoc calibration, cumulative gains, and global interpretation techniques, such as probability calibration, cumulative gain, and SHAP. However, few studies have investigated models’ calibration and cumulative gain analysis. These techniques effectively tackle uncertainty and explainability. Calibration and cumulative gain analysis are crucial tools for evaluating the performance of predictive models, particularly in medical applications. A well-calibrated model will have predicted probabilities that are close to the actual probabilities, which is important for ensuring reliable predictions and informed clinical decision-making (71). The plot is useful in making informed decisions about selecting the best model for medical diagnosis where accurate probability estimates are crucial. Cumulative gain analysis, on the other hand, evaluates the effectiveness of a model by comparing the cumulative gain of the model against a baseline model widely used in many fields (72, 73). By incorporating calibration and cumulative gain analysis into the evaluation of predictive models, we can ensure that the models are reliable, effective, and practical for use in clinical settings. The calibration curves (Figure 9) reveal the reliability of the LR and SVM models’ probability predictions. For all datasets, the LR model’s predictions align more closely with the optimal curve, indicating superior calibration. Notably, both models demonstrate better calibration performance with follow-up datasets, suggesting improved reliability over time. To gain a more profound understanding, cumulative gain analysis helped shed light on the models’ trustworthiness (Figure 10). In the cumulative gains analysis (Figure 10), the findings imply that while both models can identify a significant proportion of high-risk EUGR cases, the LR model’s performance is generally more reliable across different datasets and over time. However, the observed drop in performance during the follow-up stage underscores the importance of continuous model evaluation and adjustment. Furthermore, the SVM model’s weaker performance suggests potential areas for model improvement, perhaps through hyperparameter tuning or additional feature engineering. In summary, our study employed calibration and cumulative gain analysis to augment the trustworthiness and interpretability of the model’s predictions across different datasets. This approach confirms that the predicted probabilities align with observed outcomes, thus providing a robust basis for making informed, data-driven decisions derived from the model’s outputs. Given that explicitly describing a black-box model remains a niche (74), our study employed a global interpretable ML models to construct a decision support system, especially for making critical medical decisions. Although preterm infant prediction models that utilize ML have been previously reported (32, 34, 75), longitudinal studies involving the comparative evaluation of ML methods in an interpretable approach have been limited. Due to dynamic nature of infant growth, infants undergo rapid growth and development within their first few weeks of life. As their weight and length change significantly during this period, it is crucial to consider these changes in terms of features contribution to accurately classify and predict their growth patterns. We hypothesized that the importance of specific features might change over time as infants grow and develop. We believe that analyzing these pattern changes longitudinally will enable us to identify the most influential variables and ultimately provide a more comprehensive understanding of the data. Thus, we decided to identify which variables contribute the most to the classification of infant growth at each stage based on different outcome. Tracking changes in variable contribution from week 1 (baseline) to week 4 (follow-up) provides a longitudinal perspective, which allows for the observation of trends and patterns over time. This can help identify specific patterns and trends that may differ between populations or individual cases and lead to more effective interventions to optimize infant health and development and factors that influence infant growth, as it accounts for the dynamic nature of infant growth. In the process of developing ML models for predicting extrauterine growth restriction, both at the baseline and follow-up stages, certain variables consistently emerged as highly influential in the classification.

**Figure 11:**
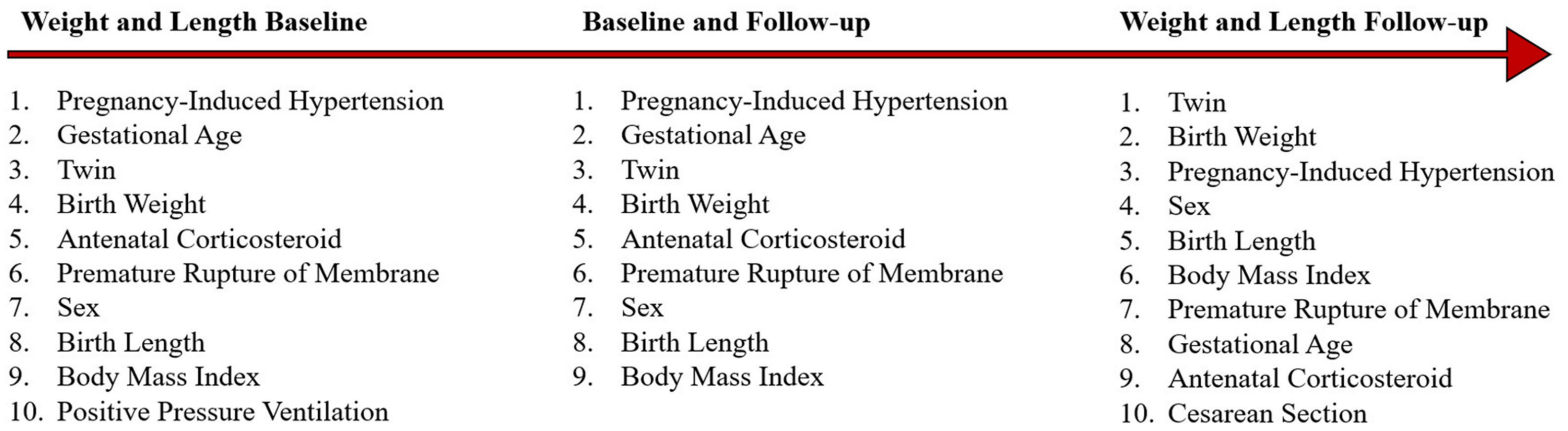
Consistency in feature importance over time.

As presented in Figure 11, in the baseline datasets, regardless of the model used (LR or SVM), Pregnancy-Induced Hypertension (PIH), Gestational Age (GA), Twin, Birth Weight (BWt), Antenatal Corticosteroid (ANC), Premature Rupture of Membrane (PROM), Sex, Birth Length (BL), Body Mass Index (BMI), and Positive Pressure Ventilation (PPV) were identified as the top ten influential variables. These variables provide a comprehensive insight into the various elements that potentially influence EUGR at birth. During the follow-up stage, the top ten influential variables included Twin, Birth Weight (BWt), Pregnancy-Induced Hypertension (PIH), Sex, Birth Length (BL), Body Mass Index (BMI), Premature Rupture of Membrane (PROM), Gestational Age (GA), Antenatal Corticosteroid (ANC), and Cesarean Section (Csec). This shift in variable importance, including the appearance of Cesarean Section in the top ten, reflects the evolving nature of growth restriction dynamics, indicating the potential impact of postnatal medical and care-related factors on EUGR progression.

Interestingly, several variables consistently ranked high in both the baseline and follow-up stages. These included pregnancy-induced hypertension, gestational age, twin, birth weight, antenatal corticosteroid, premature rupture of membrane, sex, and birth length. Their persistent significance across different stages underscores their fundamental role in the occurrence and development of EUGR. These findings emphasize the importance of a holistic approach when assessing the risk of EUGR. Multiple factors, spanning from pregnancy complications to neonatal characteristics and medical interventions, contribute to the risk and progression of EUGR. This insight can guide healthcare professionals to develop effective prevention and intervention strategies. However, these results should be interpreted with consideration of the specific dataset and model used, as the importance of each variable can vary in different contexts. We also compared the outcomes of feature importance analysis and correlation analysis to get a better grasp of the variables at play. This method not only highlighted the variables most influential to the model’s predictions but also showed how these variables relate to the target outcome in terms of direction and strength. This dual approach helps uncover intricate details in the data that could be overlooked if only one method was used.

From the analysis of SHAP feature importance and correlation analysis of four different datasets, pregnancy-induced hypertension was consistently found in the top ten features of importance in all models, as well as being in the top five positively correlated features across the four datasets. This indicates PIH is a key feature across all models and datasets, having a significant positive association with the target variable. Birth Weight and birth length are important features across all models and are also among the top five negatively correlated features in all datasets. This suggests that these features have a significant inverse relationship with the target variable, implying that as birth weight or length increases, the target variable decreases. Gestational age appeared in the top ten of the SHAP analysis for the weight baseline and was also in the top five positively correlated features for the same dataset. However, in the weight follow-up dataset, it was negatively correlated, suggesting that the relationship of GA with the target variable may vary over time. Twin status was found to be important in the weight follow-up dataset and was also positively correlated in the same dataset, indicating a significant positive relationship with the target variable in the follow-up period. Maternal body mass was found to be important and positively correlated in the weight follow-up dataset. However, in the length baseline dataset, it was negatively correlated, suggesting a complex relationship with the target variable that may vary depending on the specific context of the study.

PIH has been consistently ranked among the top ten features in all models, indicating its significant influence on the prediction models. The impact of PIH on the development of EUGR can be associated with the fact that hypertension during pregnancy can lead to restricted fetal growth (76, 77). This is a well-established clinical fact, and our models reflect this correlation, explaining the high SHAP values of PIH. Birth weight and birth length also were significant across all models. It is clinically intuitive since higher birth weight and length reduce the likelihood of growth restriction. Therefore, these factors’ high SHAP values emphasize their importance in predicting growth outcomes. In the weight baseline dataset, GA displayed a positive correlation, suggesting that lower gestational age at birth, a common risk factor for EUGR, is linked with growth restriction (78, 79). However, in the weight follow-up dataset, GA was negatively correlated. This might indicate a “catch-up” growth phenomenon, where preterm infants born at lower GA can demonstrate accelerated growth postnatally when provided with adequate nutrition and care, thereby lowering their risk of EUGR at later stages. Twin status was significant in the weight follow-up dataset and also displayed a positive correlation, reflecting the known increased risk of growth restriction in multiple pregnancies (80). Twins often face competition for nutrients *in utero*, leading to lower birth weights compared to singletons. This might result in persistent growth disparities in the postnatal period, even with adequate nutrition, hence the increased SHAP value for twin status in the follow-up dataset. Maternal body mass: The relationship between maternal body mass is associated with neonatal outcomes, which can be attributed to the multifactorial influences on fetal growth (81, 82). While higher maternal body mass often suggests better nutritional status, it can also be associated with metabolic conditions like gestational diabetes, which could impair fetal growth. This may explain the contrasting correlations in the weight follow-up and length baseline datasets.

The comparison between the results of feature importance and correlation analysis has demonstrated that feature importance and correlation are interconnected, and there are key variables, such as pregnancy-induced hypertension, birth weight, and birth length, that consistently show up as significant across different models and datasets. However, the relationship of some features with the target variable can change over time or depending on the dataset, as observed with gestational age and maternal body mass. Therefore, it’s essential to consider the context and time point when interpreting these results. This analysis has provided valuable insights into the most influential features affecting the target variable, offering a deeper understanding of the model’s behavior and potentially informing further research or interventions in this area.

The designed model in this study can be applied to clinical practice in multiple ways. First, it serves as a tool for early detection and automated classification of short-term growth outcomes in preterm infants. By providing this information, clinicians can monitor and assess the growth progress of preterm infants more effectively and promptly, thus facilitating timely and personalized interventions. Second, the model’s ability to assess and rank the importance of various features using SHAP values can help healthcare professionals understand which factors contribute most significantly to the predicted outcomes. This information can be invaluable in devising targeted treatment plans and preventive measures. Furthermore, the model can be integrated into a clinical decision support system (CDSS), providing healthcare professionals with valuable insights and recommendations based on the individual’s data. Through the CDSS, clinicians can receive risk predictions and potential growth trajectories for each infant, which can inform their decision-making process. Finally, our machine learning models can be continuously updated and refined as new patient data become available, allowing the predictive performance to improve over time. This adaptability can make it a powerful tool in managing and monitoring the growth of preterm infants.

As added merit of this study is that that our methodology diverges from the traditional longitudinal analysis for following reasons; (a) by separately analyzing baseline and follow-up datasets, we could meticulously evaluate and fine-tune our models for each specific time point. This allowed us to avoid potential biases or over-generalizations that might occur in joint analyses, (b) Enhanced interpretability by focusing on the shift in variable importance over time, our approach makes it easier for clinicians and researchers to grasp the changing dynamics of pediatric growth and EUGR risk factors. This is crucial for real-world applications where understanding the ‘why’ behind predictions is as important as the predictions themselves, (c) Diversity of data with the use of three distinct datasets ensured a broad spectrum of data, capturing the intricacies and nuances of infant growth over a period. This adds depth and richness to our analysis (that cannon be found in other studies), providing a more holistic view of pediatric growth trajectories, (d) Opportunity for targeted interventions, recognizing the changing significance of variables over time provides valuable insights for timely and specific interventions. For instance, a variable that’s highly significant in week 1 but not in week 4 might suggest early-stage intervention strategies, (e) Foundation for future studies by pioneering this unique approach, our study can serve as a reference point for future research. Researchers can further explore the implications of changing variable significance over time, potentially unveiling novel insights into pediatric care.

While the results of this study are promising, there are several limitations and weaknesses that should be considered. Firstly, the study was conducted using data from a single medical center in South Korea, which may not be representative of other populations or healthcare settings. Therefore, caution should be taken when generalizing the findings to other contexts. Secondly, the study only included infants with a gestational age of less than 32 weeks, which limits the generalizability of the results to preterm infants with a gestational age greater than 32 weeks. Thirdly, the study did not include data on certain variables that may impact infant growth, such as feeding patterns and nutrient intake, which may limit the accuracy of the models. Fourthly, Considering the characteristics of our dataset and its relatively small size, it was crucial for us to carefully select a model to avoid overfitting. We conducted preliminary tests using advanced models like XGBoost, which raised concerns regarding this issue. Specifically, when analyzing the importance of features in our baseline (Supplementary Figure S5) and follow-up (Supplementary Figure S6) datasets using XGBoost, we found that more than one-third of variables had no impact on the model’s decision-making by presenting 0 values in their feature ranking. These results raised doubts about the reliability and meaningful interpretability of the model’s decisions, especially when a significant portion of features appeared to have no influence. These findings suggested that despite its seemingly high accuracy, the model might not truly reflect the underlying structure of the data and could provide misleading interpretations. Guided by these observations and our commitment to providing consistent and trustworthy insights, we made a deliberate choice to avoid complex models like boosting and deep learning. Instead, we opted for simpler yet reliable models such as SVM, and LR. While this approach may slightly increase computational complexity, it reflects our priority of emphasizing robust interpretability over mere accuracy.

Overall, while the study provides valuable insights into the use of machine learning for predicting EUGR in preterm infants, further research is needed to validate these findings in diverse populations and healthcare settings and to address the limitations of the study. The strengths of this study include the generation of four datasets and our experimental results demonstrating that all datasets can effectively predict and differentiate cases of EUGR and non-EUGR. Furthermore, the weight-based datasets provide higher prediction performance than length-based datasets, and the follow-up datasets outperformed the baseline datasets as they exhibited greater differentiation abilities.

## 6 Conclusion

We presented a workflow to extend ML models to EUGR classification and achieved high accuracy for two-scheme classification across four datasets. We developed and validated interpretable ML predictive models for EUGR classification from prospective longitudinal clinical data. In general, ML tools have the potential to aid in the early diagnosis and treatment of infants with inadequate growth. The dynamic nature of infant growth and the critical role of tracking changes in weight and length over time necessitate a holistic approach when assessing the risk of EUGR. Monitoring shifts in variable contribution from week 1 (baseline) to week 4 (follow-up) offers a longitudinal perspective, allowing the observation of trends and patterns over time, thus providing a more comprehensive understanding of the data. These findings carry important implications for the development of effective prevention and intervention strategies for infants at risk of inadequate growth. The application of our machine learning model to clinical practice can serve as a potent tool for the early detection and classification of growth outcomes in preterm infants, enabling more effective monitoring and facilitating timely intervention. The use of global interpretation further aids clinicians by highlighting crucial contributing factors to the predicted outcomes, thereby helping in devising targeted treatment plans. The possibility of integrating these models into a clinical decision support system presents an opportunity for personalized and dynamic care, providing healthcare professionals with valuable insights and recommendations. However, the significance of specific variables may fluctuate depending on the dataset and model used, underscoring the necessity for careful interpretation of these results. Overall, this study exhibits the potential of ML tools to aid in the early diagnosis and treatment of infants with inadequate growth, offering valuable insights for healthcare professionals aiming to develop efficacious prevention and intervention strategies.

## Data availability statement

The data supporting this study’s findings are available from the corresponding author upon reasonable request. The code used in this study is available from the corresponding author upon reasonable request (https://github.com/payam-kassani).

## Ethics statement

The studies involving humans were approved by the Institutional Review Board of the CHA Bundang Medical Center (BD2015-223). The studies were conducted in accordance with the local legislation and institutional requirements. Written informed consent for participation in this study was provided by the participants’ legal guardians/next of kin. Written informed consent was obtained from the individual(s), and minor(s)’ legal guardian/next of kin, for the publication of any potentially identifiable images or data included in this article.

## Author contributions

KC: data curation, conceptualization, conceived and designed the methodology, and writing the original draft. PK: conceptualization, carried out all the analysis and experiments methodology, writing the original draft, and supervision. EK, JK, and J-WJ: analyzed and discussed the results and investigation. C-HY: review and editing and investigation. HJ: project administration, resources, funding acquisition, supervision, and review and editing. All authors contributed to the article and approved the submitted version.

## Funding

This research received no specific grant from any funding agency in the public, commercial, or not-for-profit sectors.

## Supplementary material

### SUPPLEMENTARY FIGURE S1

SHAP feature importance measured as the mean absolute Shapley values for weight baseline dataset. **(A)** Logistic regression **(B)** support vector machine.

### SUPPLEMENTARY FIGURE S2

SHAP feature importance measured as the mean absolute Shapley values for length baseline dataset. **(A)** Logistic regression **(B)** support vector machine.

### SUPPLEMENTARY FIGURE S3

SHAP feature importance measured as the mean absolute Shapley values for weight follow-up dataset. **(A)** Logistic regression **(B)** support vector machine.

### SUPPLEMENTARY FIGURE S4

SHAP feature importance measured as the mean absolute Shapley values for length follow-up dataset. **(A)** Logistic regression **(B)** support vector machine.

### SUPPLEMENTARY FIGURE S5

SHAP feature importance measured as the mean absolute Shapley values with XGBoost model in baseline datasets. **(A)** Weight baseline dataset **(B)** length baseline dataset

### SUPPLEMENTARY FIGURE S6

SHAP feature importance measured as the mean absolute Shapley values with XGBoost model in follow-up datasets. **(A)** Weight follow-up dataset **(B)** length follow-up dataset.
