# Supplementary figures and images for: Comparative effectiveness of explainable machine learning approaches for extrauterine growth restriction classification in preterm infants using longitudinal data
Source: Front Med (Lausanne). 2023 Nov 29;10:1166743. doi: 10.3389/fmed.2023.1166743 (PMC10716334; doi:10.3389/fmed.2023.1166743)

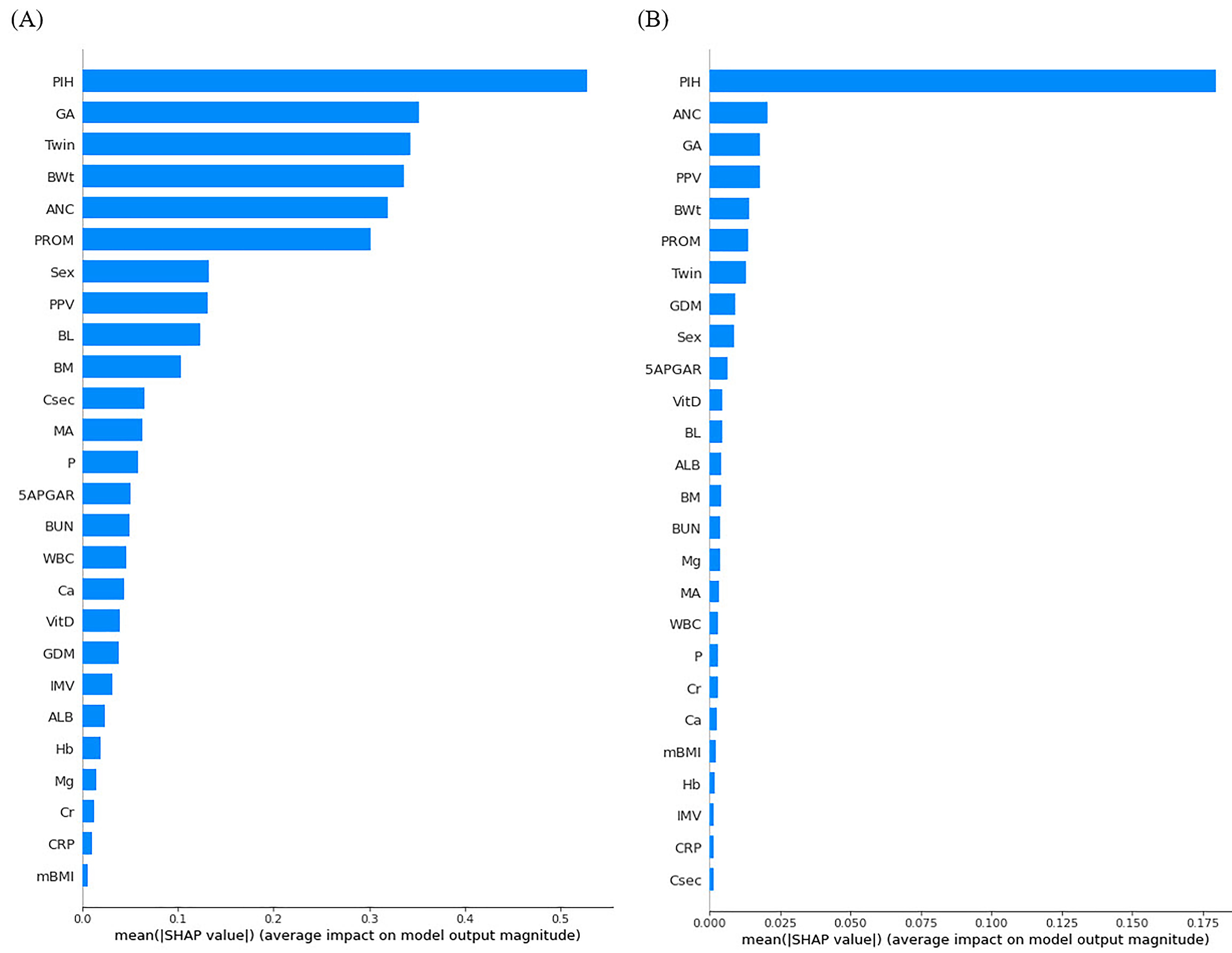

Supplement: Supplementary file 1 [file Image_1.jpeg]

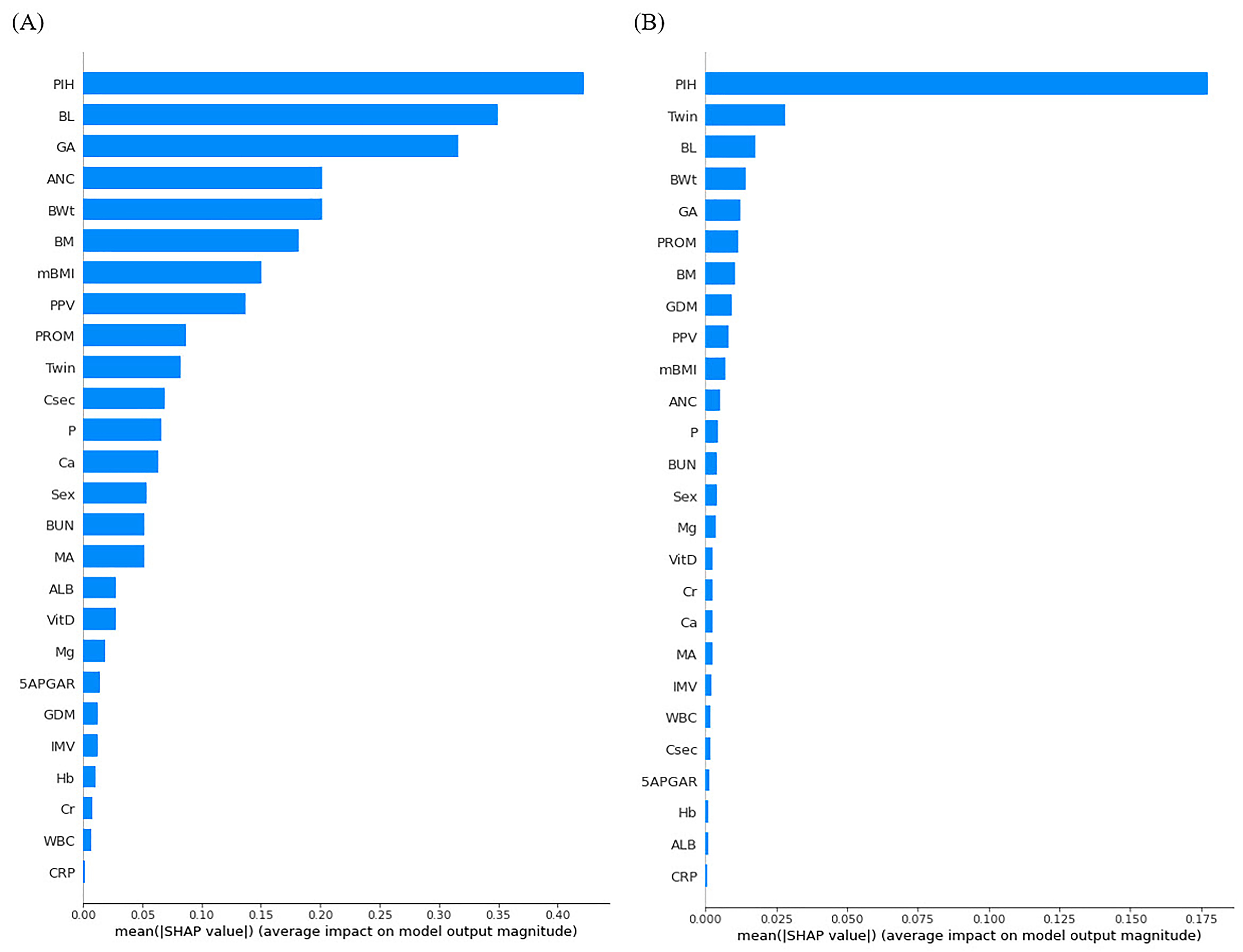

Supplement: Supplementary file 2 [file Image_2.jpeg]

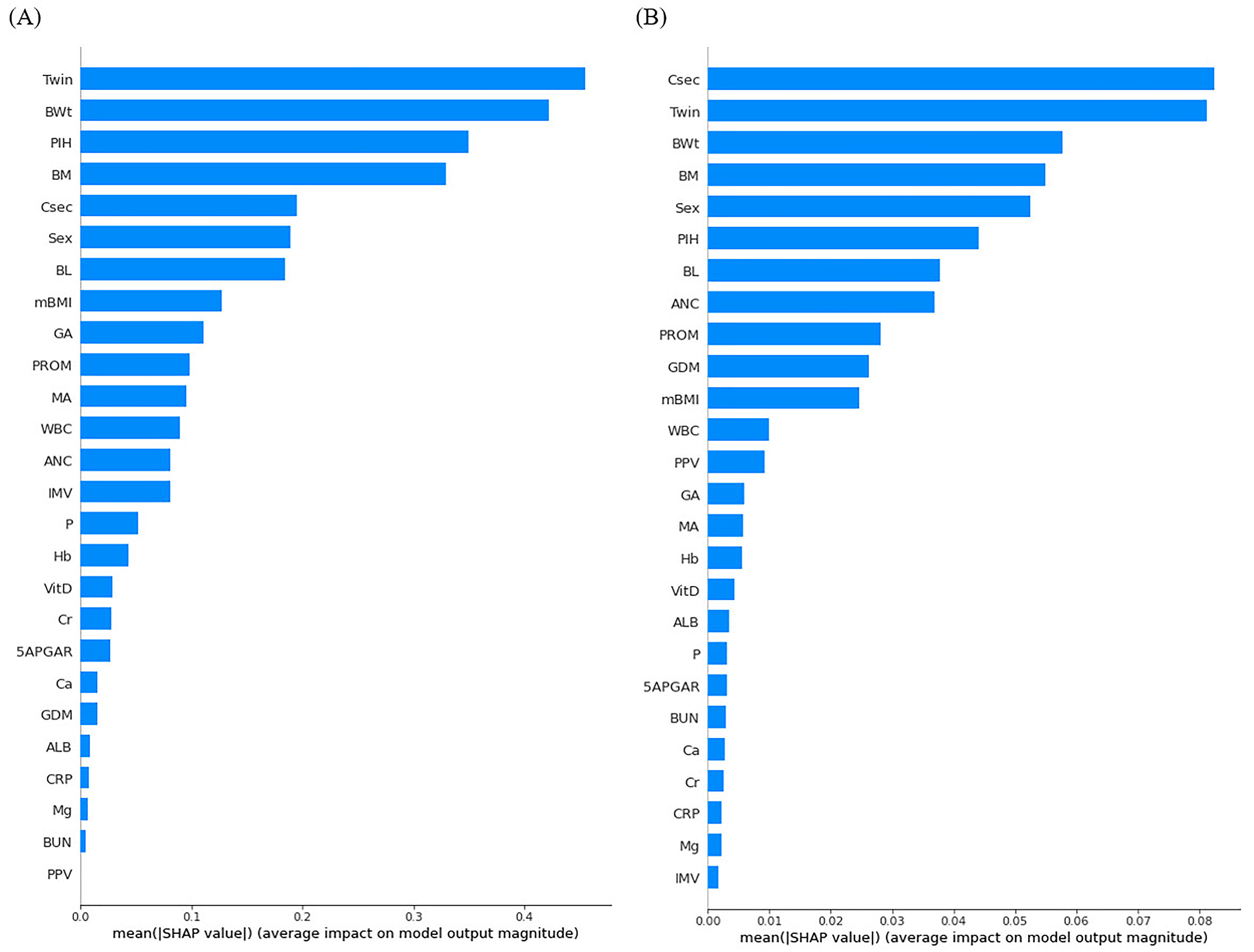

Supplement: Supplementary file 3 [file Image_3.jpeg]

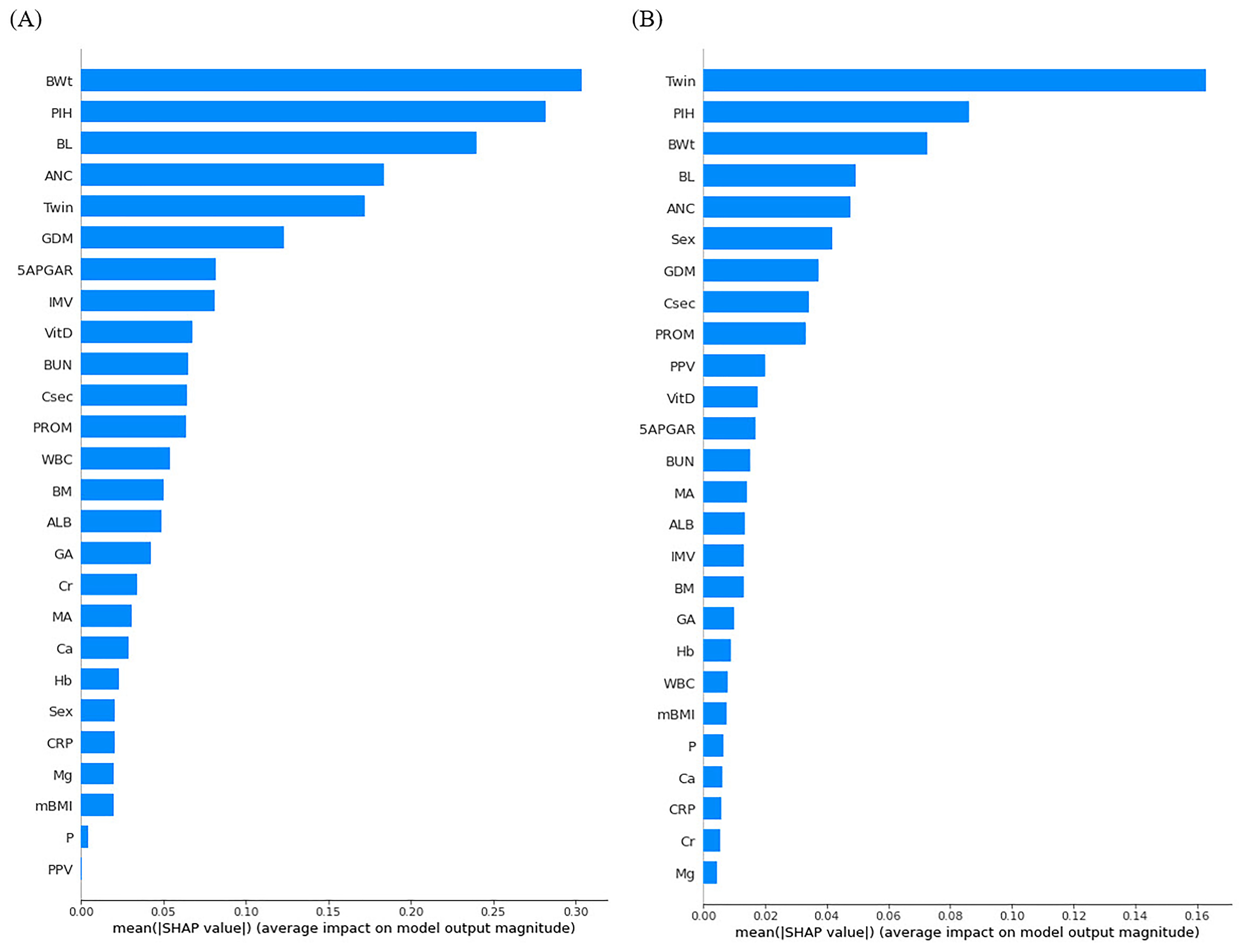

Supplement: Supplementary file 4 [file Image_4.jpeg]

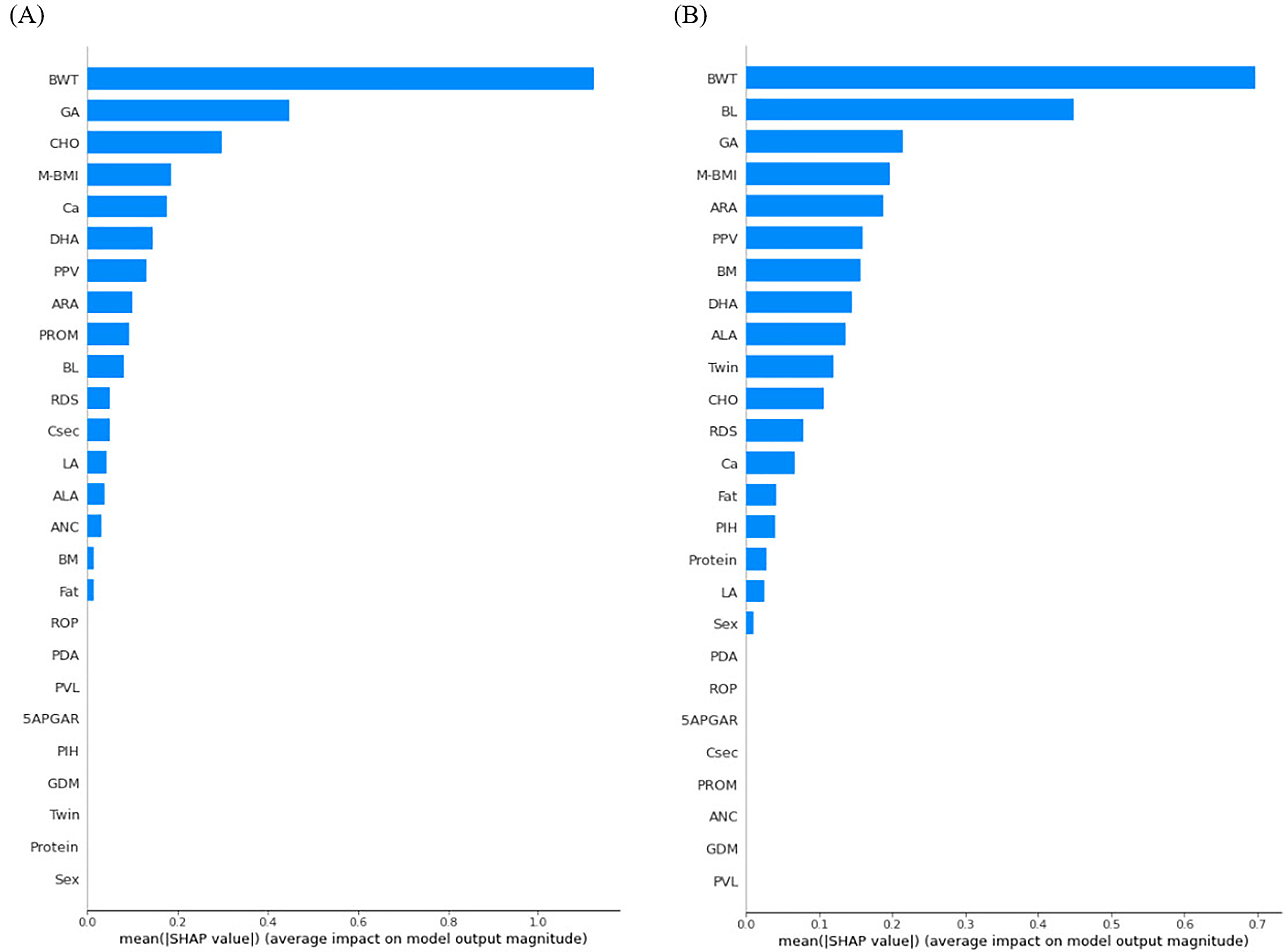

Supplement: Supplementary file 5 [file Image_5.jpeg]

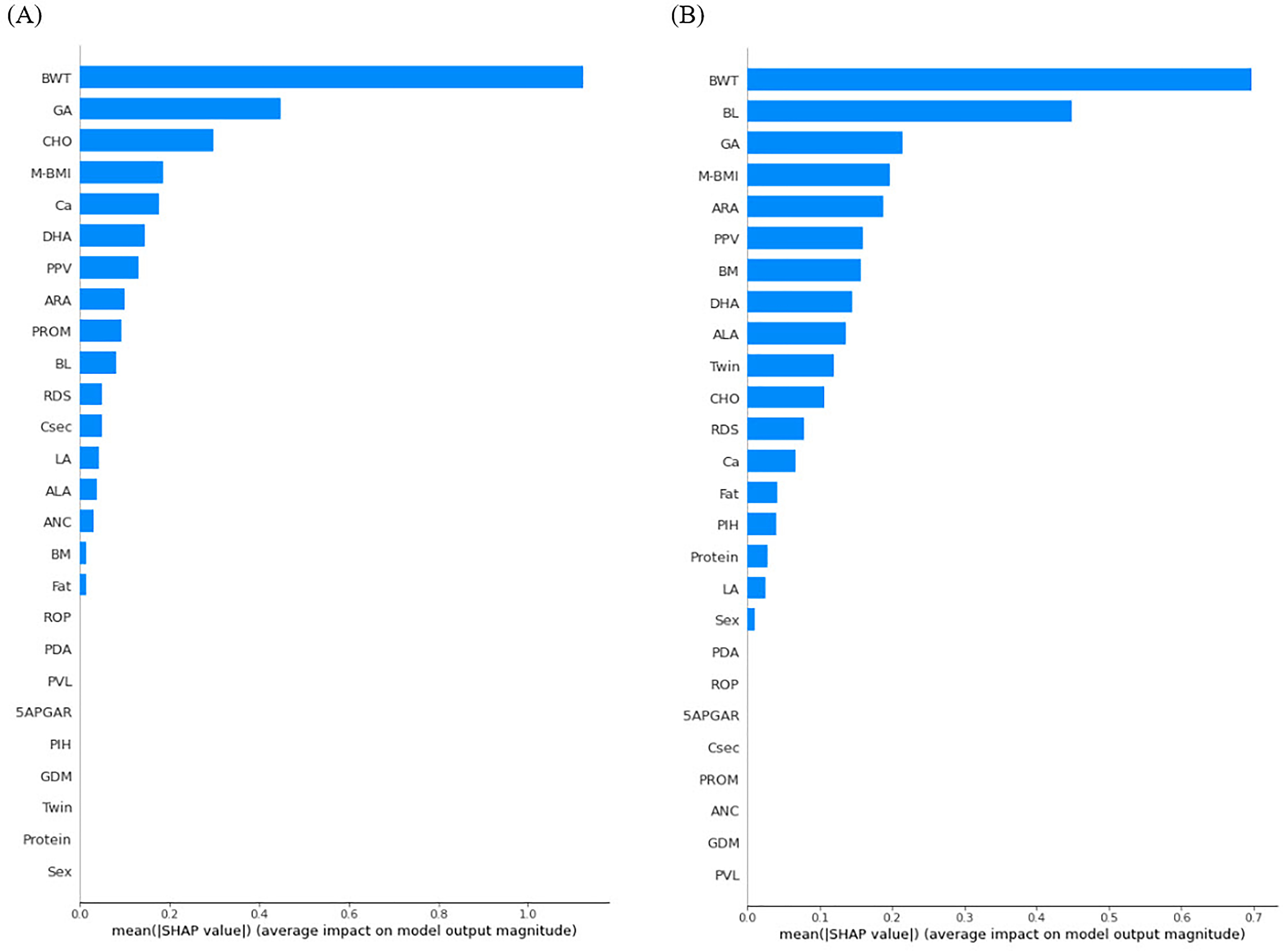

Supplement: Supplementary file 6 [file Image_6.jpeg]
